# Mg chelatase in chlorophyll synthesis and retrograde signaling in *Chlamydomonas reinhardtii*: CHLI2 cannot substitute for CHLI1

**DOI:** 10.1093/jxb/erw004

**Published:** 2016-01-25

**Authors:** Pawel Brzezowski, Marina N. Sharifi, Rachel M. Dent, Marius K. Morhard, Krishna K. Niyogi, Bernhard Grimm

**Affiliations:** ^1^Institute of Biology/Plant Physiology, Humboldt University, Philippstraße 13, D-10115 Berlin, Germany; ^2^Department of Plant and Microbial Biology, Howard Hughes Medical Institute, University of California, Berkeley, CA 94720-3102, USA; ^3^Physical Biosciences Division, Lawrence Berkeley National Laboratory, Berkeley, CA 94720, USA

**Keywords:** *Chlamydomonas*, CHLI, chloroplast retrograde signaling, Mg chelatase, protoporphyrin IX, tetrapyrrole biosynthesis pathway.

## Abstract

The oligomeric Mg chelatase (MgCh), consisting of the subunits CHLH, CHLI, and CHLD, is located at the central site of chlorophyll synthesis, but is also thought to have an additional function in regulatory feedback control of the tetrapyrrole biosynthesis pathway and in chloroplast retrograde signaling. In *Arabidopsis thaliana* and *Chlamydomonas reinhardtii*, two genes have been proposed to encode the CHLI subunit of MgCh. While the role of CHLI1 in *A. thaliana* MgCh has been substantially elucidated, different reports provide inconsistent results with regard to the function of CHLI2 in Mg chelation and retrograde signaling. In the present report, the possible functions of both isoforms were analyzed in *C. reinhardtii*. Knockout of the *CHLI1* gene resulted in complete loss of MgCh activity, absence of chlorophyll, acute light sensitivity, and, as a consequence, down-regulation of tetrapyrrole biosynthesis and photosynthesis-associated nuclear genes. These observations indicate a phenotypical resemblance of *chli1* to the *chlh* and *chld C. reinhardtii* mutants previously reported. The key role of CHLI1 for MgCh reaction in comparison with the second isoform was confirmed by the rescue of *chli1* with genomic *CHLI1*. Because *CHLI2* in *C. reinhardtii* shows lower expression than *CHLI1*, strains overexpressing CHLI2 were produced in the *chli1* background. However, no complementation of the *chli1* phenotype was observed. Silencing of *CHLI2* in the wild-type background did not result in any changes in the accumulation of tetrapyrrole intermediates or of chlorophyll. The results suggest that, unlike in *A. thaliana*, changes in CHLI2 content observed in the present studies do not affect formation and activity of MgCh in *C. reinhardtii*.

## Highlight

CHLI1 is indispensable for MgCh function in *C. reinhardtii*, while CHLI2 is not involved in MgCh activity. Inactivation of MgCh due to CHLI1 deficiency results in altered nuclear gene expression.

## Introduction

The first committed enzyme in the chlorophyll branch of tetrapyrrole biosynthesis (TBS) in photosynthetic organisms is Mg chelatase (MgCh). MgCh is responsible for the catalysis of Mg^2+^ insertion into protoporphyrin IX (Proto), which results in formation of Mg protoporphyrin IX (MgProto). MgCh is a heterotrimeric complex consisting of least three separable proteins, CHLI (36–46kDa), CHLD (60–87kDa), and CHLH (120–155; Walker and Weinstein, 1991; Willows *et al.*, 1996).

In the vascular plant *Arabidopsis thaliana* and in the green alga *Chlamydomonas reinhardtii*, one gene encodes the CHLD subunit, while two genes each for the CHLH and CHLI subunits have been identified. Based on the annotations and sequences obtained from the *C. reinhardtii* genome v5.5, Phytozome v10.2, expression of all these genes is supported by multiple expressed sequence tags (ESTs) in the dbEST of GenBank (http://www.ncbi.nlm.nih.gov/genbank, accessed 15 January 2016). The presence of two genes for CHLH and CHLI points to the possibility of different functions of the two isoforms of these proteins. However, despite the sizeable amount of research on MgCh, the exact role of each of its intrinsic subunits still remains elusive. CHLI contains Mg-ATP-binding motifs (Hansson *et al.*, 2002), and the ATPase activity of CHLI was demonstrated *in vitro* (Sirijovski *et al.*, 2006). CHLH was shown to bind Proto (Willows *et al.*, 1996), while CHLD is thought to provide a platform for the assembly of the CHLI subunits (Sirijovski *et al.*, 2006).

Previous analysis in *A. thaliana* indicated that the transcript levels of *CHLI1* and *CHLI2* are similar, but because CHLI2 protein could not be detected in the *chli1* mutant, it was assumed that it undergoes a rapid post-translational turnover (Rissler *et al.*, 2002). However, other studies in *A. thaliana* demonstrated that *CHLI2* is expressed at a much lower level than *CHLI1* (Brenner *et al.*, 2000; Matsumoto *et al.*, 2004; Huang and Li, 2009). It was also suggested that CHLI2 cannot be assembled into the hexameric ring because of changes in the residues of its C-terminus (Apchelimov *et al.*, 2007). It was concluded that the function of CHLI2 cannot be determined due to its low expression level. Nevertheless, the recombinant CHLI2 of *A. thaliana* has an ATPase activity, although with a lower maximum reaction rate (*V*_max_) and higher Michaelis constant (*K*_mATP_) than CHLI1, and it shows thioredoxin-dependent reduction of a disulfide bond and thiol modulation of its ATPase activity (Kobayashi *et al.*, 2008). However, in more recent work, it was demonstrated that overexpression of the native CHLI2 in *A. thaliana* can complement impairment caused by the mutation in *CHLI1* (Huang and Li, 2009).

The chloroplast can exert changes on nuclear gene expression (Beck, 2001). A series of experiments led to the discovery of the *genomes uncoupled* (*gun*) mutants, which showed disturbed retrograde signaling, resulting in deregulation of nuclear gene expression (Susek *et al.*, 1993). Inactivation of MgCh due to the mutation in CHLH was demonstrated in the *A. thaliana gun5* mutant (*genomes uncoupled 5*), resulting in deregulated expression of the *LHCB2* gene (Mochizuki *et al.*, 2001). Interestingly, *chlh* and *chld* mutants characterized with deficient MgCh activity show plastid-mediated deregulation of selected nuclear genes (Chekunova *et al.*, 2001; von Gromoff *et al.*, 2008; Brzezowski *et al.*, 2014).

Although it was reported that *A. thaliana cs* and *ch42* mutants with defects in CHLI1 do not show the *gun* phenotype [modified gene expression in the presence of norflurazon (NF) in comparison with NF-treated wild-type seedlings (Mochizuki *et al.*, 2001)], it was suggested that semi-dominant *cs215/cs215* and the *chli1/chli1 chli2/chli2* double mutant do, because they accumulate a higher level of *LHCB1* transcript than the wild type upon NF treatment (Huang and Li, 2009).

Different studies correlated the product of MgCh activity with its function in chloroplast retrograde signaling, suggesting that MgProto is required for down-regulation of nuclear gene expression by means of transmitting the signal from the chloroplast (Johanningmeier and Howell, 1984; Susek *et al.*, 1993; Oster *et al.*, 1996; Kropat *et al.*, 1997, 2000; La Rocca *et al.*, 2001; Strand *et al.*, 2003). Although the involvement of Mg porphyrins in chloroplast retrograde signaling was questioned in several studies (Mochizuki *et al.*, 2008; Moulin *et al.*, 2008; Brzezowski *et al.*, 2014; Schlicke *et al.*, 2014), it still seems to be a matter of debate (Zhang *et al.*, 2011; Zhang *et al.*, 2015).

The functions of the two CHLI isoforms in Mg chelation of Proto and the possible effect of disturbed tetrapyrrole biosynthesis on plastid-derived retrograde signaling were examined in the present study. The substantial role of CHLI1 in the MgCh reaction has been demonstrated, while overexpression or silencing of *CHLI2* led to the conclusion that the second CHLI isoform in *C. reinhardtii* is apparently not necessary for MgCh activity. With regard to the effect of the *chli1* knockout on chloroplast retrograde signaling, down-regulation of several tetrapyrrole biosynthesis-related and photosynthesis-associated nuclear (PhAN) genes was observed.

## Materials and methods

### Strains and culture conditions

Because of the acute light sensitivity (Fig. 2B; Supplementary Fig. S5 at *JXB* online) and a complete lack of chlorophyll (Supplementary Fig. S2A, B), the *chli1/fdx3* mutant was maintained on agar-solidified TRIS-acetate-phosphate (TAP; Gorman and Levine, 1965; Harris, 1989) in the dark, at 23 °C. Genetic crosses were performed as described previously (Harris, 1989).

The *CC-3395* cell wall-deficient (*cwd*) arginine auxotrophic (*arg7-8*), mating-type minus (mt–) strain was obtained from the Chlamydomonas Center (University of Minnesota, St. Paul, MN, USA). *CC-3395* carries a mutation in argininosuccinate lyase (*ARG7*) and was maintained on agar-solidified TAP supplemented with 5 µg ml^−1^ arginine at 50 µmol photons m^−2^ s^−1^ and 23 °C. In the present study, both *CC-3395* and 4A+ were referred to as the wild-type strains.

### Analysis of tetrapyrrole intermediates and end-products

Samples from all the strains, normalized to contain 1.2×10^8^ cells, were collected by centrifugation. Pellets were snap-frozen in LN_2_, followed by extraction with acetone/0.2M NH_4_OH (9/1, v/v) cooled to –20 °C to prevent chlorophyll degradation by chlorophyllase (Hu *et al.*, 2013). The pellets obtained were used for the extraction of heme with acetone/HCl/DMSO (10/0.5/2, v/v/v) at room temperature. TBS intermediates and end-products were analyzed by HPLC (Czarnecki *et al.*, 2011).

### Preparation of nucleic acids and transcript analyses

The total DNA, used as a template for amplification of genomic *CHLI1* and *FDX3* for the rescue transformation of *chli1/fdx3*, was isolated from 4A+ as described previously (Brzezowski *et al.*, 2012).

Total RNA was isolated using TRIzol Reagent (Life Technologies, http://www.lifetechnologies.com, accessed 15 January 2016) according to the manufacturer’s instruction. For both reverse transcription–PCR (RT–PCR) and quantitative RT–PCR (qRT–PCR), the constitutively expressed *18S* rRNA was used as the control using PB69 and PB70 primers (Supplementary Table S2). Primers for RT–PCR and qRT–PCR were designed using PRIMER3PLUS (http://www.bioinformatics.nl/cgi-bin/primer3plus/primer3plus.cgi, accessed 15 January 2016; Supplementary Table S2). Transcripts for *CHLI1*, *CHLI2*, and *CHLI1*-neighboring genes were analyzed by RT–PCR in cells from liquid TAP media kept in the dark (Supplementary Fig. S1). Transcript analyses of the genes encoding enzymes of the TBS pathway and PhAN genes (Supplementary Fig. S3) were examined by qRT–PCR in the dark and following exposure to 20 µmol photons m^−2^ s^−1^ light, for 1, 2, 4, and 8h. The *CHLI2* transcripts in strains overexpressing *CHLI2* were examined in the dark, while transcripts of *CHLI1*, *CHLI2*, and *GPX5* were analyzed in cells exposed to 50 µmol photons m^−2^ s^−1^ light.

### Vector construction

Two vectors were constructed for the attempts to rescue *chli1/fdx3*. The pMS188 plasmid (Schroda *et al.*, 2002) carries a bleomycin resistance cassette (BleR; Stevens *et al.*, 1996). The 4282bp long genomic *CHLI1* was PCR amplified using primers PB207 and PB208 with *Kpn*I and *Psi*I restriction sites attached, respectively (Supplementary Table S2) and was introduced into pMS188 pre-digested with *Kpn*I/*Psi*I to generate the CHLI1/pMS188 plasmid (Supplementary Fig. S4A). The primers PB209 and PB210, with *Psi*I and *Dra*III attached, respectively (Supplementary Table S2), were used to amplify a 1556bp genomic *FDX3* fragment. The DNA was introduced into the pMS586 vector (pHyg3; obtained from Michael Schroda, University of Kaiserslautern, Germany) pre-digested with *Psi*I/*Dra*III, resulting in FDX3/pMS586 (Supplementary Fig. S4A). Transformation of *chli1/fdx3* was performed with CHLI1/pMS188 or FDX3/pMS586, or both plasmids at the same time. Selection was conducted on 15 µg ml^−1^ zeocin, 10 µg ml^−1^ hygromycin, or both antibiotics, depending on the plasmid used. Following transformation and recovery in the dark, all plates were transferred to 50 μmol photons m^−2^ s^−1^ to increase the selection pressure.

The *CHLI2* coding sequence (CDS) was amplified by PCR from cDNA using forward primers PB317 and PB313 with *Nde*I and *Nae*I restriction sites attached, respectively, and the reverse primer PB314 carrying an *Eco*RI site (Supplementary Table S2), followed by ligation into pGenD2 (Fischer and Rochaix, 2001) pre-digested with *Nde*I and *Eco*RI or *Nae*I and *Eco*RI, producing pCHLI2/GenD2 without and pCHLI2/GenD2-cTP with the CDS of the PSAD transit peptide (Supplementary Fig. S4B). Both vectors were used for co-transformation of the *chli1/fdx3* strain with pMS188, and selection was conducted on TAP-agar plates with 15 µg ml^−1^ zeocin with illumination of 10 µmol photons m^−2^ s^−1^.

For the silencing of *CHLI2*, the MicroRNA Designer platform (WMD3, http://wmd3.weigelworld.org/cgi-bin/webapp.cgi, accessed 15 January 2016) was used to design suitable 90-mer oligonucleotides (PB449 and PB450; Supplementary Table S2) specific for targeting the region spanning exon 9 and the 3'-untranslated region (UTR) of *CHLI2*, carrying two mismatches corresponding to positions 1410 and 1427 of *CHLI2* cDNA (genome v5.5, Phytozome v10.2; Supplementary Table S2). Following annealing of PB449 and PB450 (Supplementary Table S2), phosphorylation, and digestion with *Spe*I, the 90bp dsDNA was introduced into the *Spe*I site of the artificial miRNA (amiRNA) precursor cre-MIR1157 in the pChlamiRNA2 vector (Molnar *et al.*, 2009), producing CHLI2/amiRNA-E9/3'UTR, which was used for *CC-3395* transformation. Selection was based on the expression of *ARG7* in pChlamiRNA2 on TAP plates without arginine, and at 10 µmol photons m^−2^ s^−1^, to avoid a possible phototoxic effect in potentially light-sensitive transformants. All *C. reinhardtii* transformations were performed by electroporation (Shimogawara *et al.*, 1998).

### Protein analyses

The total protein content was extracted in 400 µl of buffer containing 56mM Na_2_CO_3_, 56mM DTT, 2% SDS, 12% sucrose, and 2mM EDTA. The total protein amounts were determined using a Pierce BCA Protein Assay Kit (Life Technologies). For the analysis of the CHLI1 and CHLI2 content in *chli1/fdx3* and strains overexpressing CHLI2, 50 µg of the total protein was loaded, while 10 µg was used for analysis in strains with silenced CHLI2. Proteins were separated by 12% SDS–PAGE, followed by transfer to a nitrocellulose membrane. The polyclonal CHLI1 antibody developed for *A. thaliana* was used, which recognized both CHLI1 and CHLI2 of *C. reinhardtii*. The chemiluminescence signal was detected using Stella 3200 (Raytest Isotopenmessgeräte GmbH, http://www.raytest.de, accessed 15 January 2016). Quantification of the signal was performed using AIDA/2D densitometry software (Raytest).

### *In silico* analysis of the CHLI proteins

**Fig. 1.**
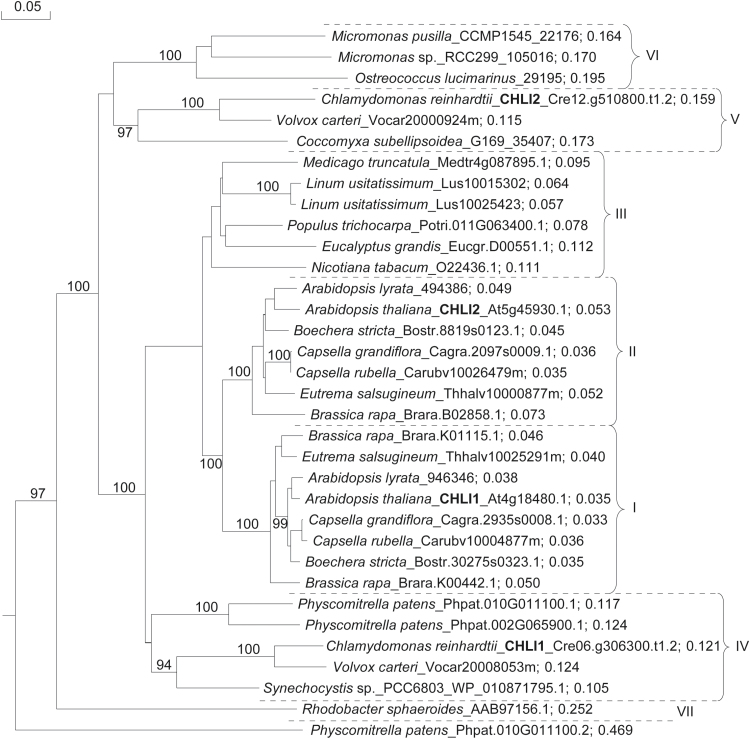
Phylogenetic tree constructed for comparative purposes, based on the alignment (Supplementary Fig. S9) of annotated CHLI amino acid sequences from selected organisms. Species, transcript names, and a sequence weight are indicated. The analysis was performed using DNAMAN, with quick alignment and based on observed divergence. Bootstrap values from 1000 iteration analysis are shown in italics; a detailed description is given in the text.

To analyze the homology between the CHLI proteins from different organisms, CHLI1 and CHLI2 sequences from *A. thaliana* and *C. reinhardtii* were used as queries within the Viridiplantae by the BLAST tool on Phytozome v10.2. The 10 highest scores for each BLAST search were selected, duplicates from different searches were removed, and transcript names were used for protein identification. Additionally, sequences for *Synechocystis* sp. PCC6803, *Rhodobacter sphaeroides*, and *Nicotiana tabacum* were obtained from the NCBI database (http://www.ncbi.nlm.nih.gov/protein/, accessed 15 January 2016), under the transcript names of WP_010871795.1, AAB97156.1, and O22436.1, respectively. The phylogenetic tree (Fig. 1) was constructed using DNAMAN software Version 6 (Lynnon Corporation, Pointe-Claire, QC, Canada). Bootstrap values were calculated from 1000 iterations; the alignment can be found in Supplementary Fig. S9.

## Results

### Isolation of a mutant lacking the *CHLI1* gene

**Fig. 2.**
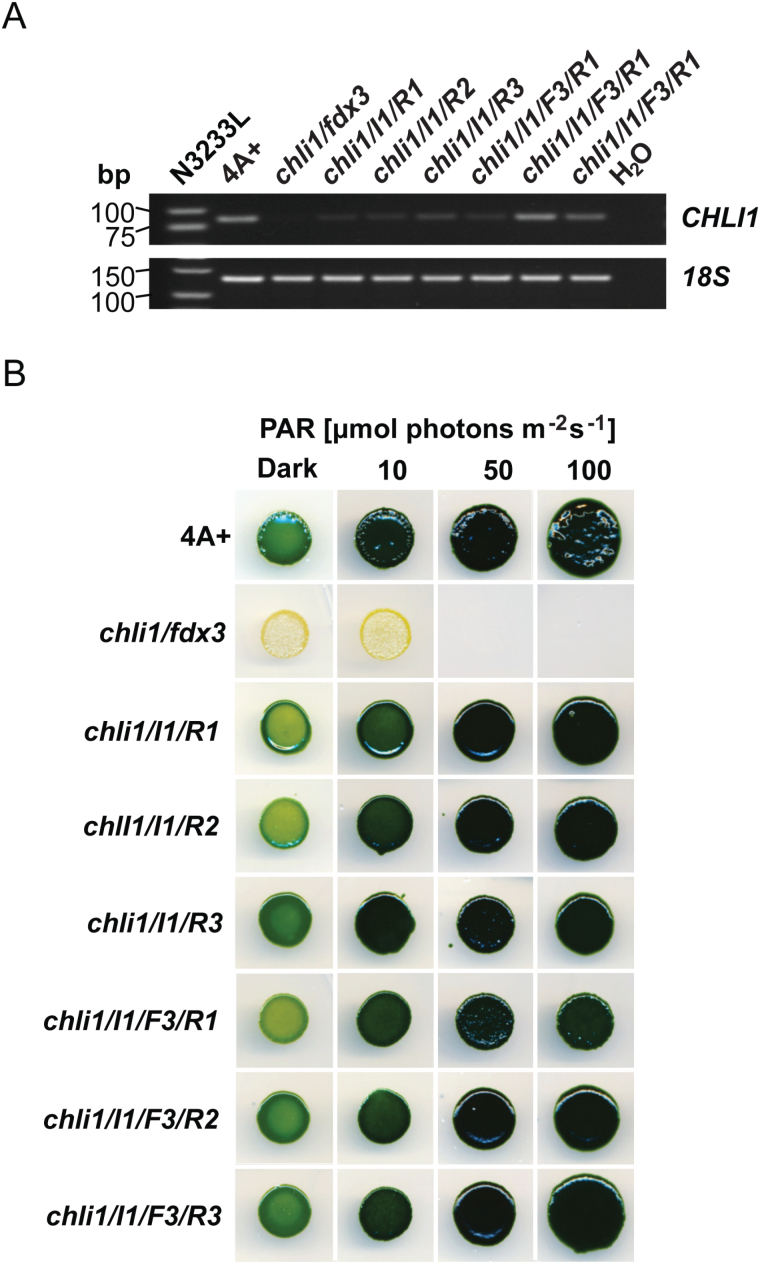
Analysis of *chli1/fdx3* and strains obtained from the rescue transformation with CHLI1/pMS188 or FDX3/pMS586, or both plasmids simultaneously. (A) The RT–PCR examination of the presence of the *CHLI1* transcripts; the low molecular weight DNA N3222L ladder (New England Biolabs GmbH, Frankfurt am Main, Germany) is shown. (B) General appearance, growth in the dark, and sensitivity to 10, 50, and 100 µmol photons m^−2^ s^−1^ of photosynthetically active radiation (PAR).

CAL029C_08 is a light-sensitive brown mutant (Fig. 2B), which was isolated in a screen for *C. reinhardtii* mutants that are unable to grow photoautotrophically (Dent *et al.*, 2015). The mutant was generated by transformation of the 4A+ wild-type strain with plasmid carrying the *aphVIII* cassette, which confers resistance to paromomycin (Sizova *et al.*, 2001). Progeny of a cross between CAL029C_08 and the wild type showed a 2:2 pattern of segregation of the brown phenotype in complete tetrads (Supplementary Table S1), demonstrating that the phenotype is due to a single nuclear mutation. Altogether, a total of 73 progeny were obtained (including progeny from incomplete tetrads), of which 39 were wild type and 34 were mutants. All the mutants and none of the wild-type progeny were resistant to paromomycin (Supplementary Table S1), indicating that the insertion of the *aphVIII* cassette is tightly linked to the mutation causing the brown phenotype.

Isolation of the genomic DNA region adjacent to the insertion site in CAL029C_08 was conducted by RESDA-PCR (Gonzalez-Ballester *et al.*, 2005), which yielded a 267bp flanking sequence on chromosome 6:8351310-8351576, upstream from *CHLI1* (genome v5.5, Phytozome v10.2). *CHLI1* mRNA was undetectable in RT–PCR analysis, demonstrating that CAL029C_08 carries a null mutation in the *CHLI1* gene (*chli1/fdx3*; Fig. 2A; Supplementary Fig. S1A). However, because insertional mutagenesis in *C. reinhardtii* often results in deletions of relatively large fragments of genomic DNA (Tam and Lefebvre, 1993; Matsuo *et al.*, 2008), the effect of the introduced exogenous DNA on *CHLI1*-neighboring genes was analyzed. Indeed, not only the mRNA of *CHLI1*, but also transcripts of several other genes located upstream or downstream of *CHLI1* were missing in the CAL029C_08 mutant (Supplementary Fig. S1B, C). Among the genes affected by the insertion was *FERREDOXIN 3* (*FDX3*, *Cre06.g306350;* genome v5.5, Phytozome v10.2; Supplementary Fig. S1B, C). Because no other gene affected by the insertion carried an annotation, the CAL029C_08 mutant strain was labeled *chli1/fdx3*.

### The *chli1/fdx3* mutant is affected in the chlorophyll branch of TBS

Analysis of the tetrapyrrole and pigment accumulation in *chli1/fdx3* in the dark revealed a >100-fold increase in Proto compared with the wild type (Supplementary Fig. S2A; Table 1). Remarkably, there was no detectable MgProto and chlorophyll in *chli1/fdx3*. The MgProto steady-state level in the wild type was low, but detectable, probably due to the lower chlorophyll synthesis in the dark compared with light conditions (Supplementary Fig. S2). Moreover, lower levels of violaxanthin, antheraxanthin, neoxanthin, lutein, and β-carotene were detected (Supplementary Figs S2A, S6D–F, H, I), while higher levels of zeaxanthin were observed in the mutant compared with the wild type (Supplementary Figs S2A, B, S6G). Although, antheraxanthin was always lower in *chli1/fdx3* in the dark and at 4h in the light, compared with the wild type (Supplementary Figs S2A, S6F), exposure to light for 24h caused its increase in *chli1/fdx3*, with values exceeding the levels observed in the wild type (Supplementary Fig. S2B).

**Table 1.** Accumulation of TBS intermediates and chlorophyll in dark conditions, in *chli1/fdx3* and the rescued lines, obtained from the transformation with CHLI1/pMS188 (*I1/R-1*, *I1/R-2*, and *I1/R-3*) or both CHLI1/pMS188 and FDX3/pMS586 (*I1/F3/R-1*, *I1/F3/R-2*, and *I1/F3/R-3*).

	Proto	Chl *a*	Chl *b*
**4A+**	0.004±0.001 (1.0)	2440.5±27.6 (1.0)	1344.0±19.6 (1.0)
***chli1/fdx3***	0.565±0.260 (145.2)	0	0
***I1/R-1***	0.002±0.002 (0.6)	786.4±7.2 (0.32)	448.8±6.3 (0.3)
***I1/R-2***	0.010±0.009 (2.5)	81.6±108.0 (0.03)	69.9±121.1 (0.1)
***I1/R-3***	0.096±.148 (24.8)	317.5±6.4 (0.13)	242.1±5.6 (0.2)
***I1/F3/R-1***	0.138±0.011 (35.4)	615.1±2.7 (0.25)	378.6±3.4 (0.3)
***I1/F3/R-2***	0.009±0.005 (2.3)	844.8±24.4 (0.35)	620.2±28.2 (0.5)
***I1/F3/R-3***	0.027±0.029 (6.9)	332.7±25.3 (0.14)	182.7±44.2 (0.1)

The experiment was performed in biological triplicates; results are presented as mean values ±SD.

Amounts are given in pmol 10^–6^ cells.

Numbers in parentheses indicate relative fold difference compared with the wild-type levels (4A+).

Accumulation of selected tetrapyrrole intermediates was also analyzed after pre-growth in the dark and exposure to 20 μmol photons m^−2^ s^−1^ for 24h. It is noteworthy that almost a 15 000-fold increase in Proto was observed in *chli1/fdx3* compared with the wild type (Supplementary Fig. S2B). The *chli1/fdx3* strain also showed a complete lack of chlorophyll during illumination (Supplementary Fig. S2B). Interestingly, heme accumulated to almost 5.3 times higher levels in *chli1/fdx3* compared with the wild type (Supplementary Fig. S2C), which could be indicative of a passive or active mechanism redirecting excess Proto towards the heme branch. Violaxanthin and β-carotene levels reached nearly 24% and 20% of the wild-type level, respectively (Supplementary Fig. S2B). Following 24h exposure to 20 µmol photons m^−2^ s^−1^, 2-fold and >5-fold increases were observed in antheraxanthin and zeaxanthin in *chli1/fdx3* compared with the wild type, respectively (Supplementary Fig. S2B).

### Expression of the selected genes encoding components of TBS in *chli1/fdx3*

Lack of the functional MgCh in *C. reinhardtii chlh* and *chld* mutants results in the absence of chlorophyll and altered HSP70A expression (Chekunova *et al.*, 2001; von Gromoff *et al.*, 2008). Thus, to elucidate further the consequences of abolished chlorophyll synthesis at the level of Mg chelation on TBS-derived retrograde signaling, the expression kinetics of selected genes were analyzed by qRT–PCR in *chli1/fdx3* in the dark and upon transfer from the dark to 20 μmol photons m^−2^ s^−1^ light after 1, 2, 4, and 8h (Supplementary Fig. S3). All of the examined TBS genes, including the five genes for MgCh subunits, showed reduced light-induced expression in *chli1/fdx3* compared with the wild type. Expression of two CHLI-encoding genes in the wild type revealed a >4-fold higher expression of *CHLI1* than *CHLI2* at the 4h time point in the light (Supplementary Fig. S3L). In the wild type, *CHLI2* showed 4.4-fold lower transcript levels than *CHLI1* after 4h of light exposure (Supplementary Fig. S3L). Notably, *FeC* encoding ferrochelatase (FeCh) showed an increased expression after 1h and 2h of illumination in *chli1/fdx3* compared with the wild type (Supplementary Fig. S3O), which may explain the increased heme levels in *chli1/fdx3* exposed to light (Supplementary Fig. S2C). Genes encoding proteins of the light-harvesting complexes (LHCs) showed a reduced expression in *chli1/fdx3* compared with the wild type (Supplementary Fig. S3P–R), which indicates, as in *A. thaliana*, that knockout of *CHLI1* results in retrograde signaling-dependent modulation of nuclear gene expression in response to a complete hindrance in Mg chelation and Proto accumulation.

### The *chli1* knockout is responsible for the chlorophyll deficiency phenotype in the *chli1/fdx3* strain

To determine if the phenotypes of *chli1/fdx3* are due to mutation of *CHLI1* and/or *FDX3*, we performed transformations using genomic versions of wild-type *CHLI1* and *FDX3* in the CHLI1/pMS188 and FDX3/pMS586 vector, respectively (Supplementary Fig. S4A). Transformants that rescued the mutant phenotype were recovered when the CHLI1/pMS188 vector was used with or without FDX3/pMS586, whereas no rescued colonies were obtained from the transformation with FDX3/pMS586 alone. Thus, the *CHLI1* deletion is responsible for the phenotype of *chli1/fdx3*. Three out of 258 colonies obtained from transformation with CHLI1/pMS188 were selected and labeled *chli1/I1/R1*, *chli1/I1/R2*, and *chli1/I1/R3*. Transformation with both CHLI1/pMS188 and FDX3/pMS586 yielded 166 colonies, from which three were labeled *chli1/I1/F3/R1*, *chli1/I1/F3/R2*, and *chli1/I1/F3/R3*. The RT–PCR confirmed the *CHLI1* transcript present in all of the analyzed rescued lines, although with certain variations with regard to the accumulated mRNA levels (Fig. 2A).

Examination of growth and light sensitivity demonstrated that *chli1/fdx3* cannot survive in light conditions ≥50 μmol photons m^−2^ s^−1^ on heterotrophic medium (TAP; Fig. 2B; Supplementary Fig. S5A) and on photoautotrophic medium regardless of the light conditions (TP; Supplementary Fig. S5B). All the rescued lines showed greening in the dark and light. Moreover, lack of light sensitivity, as observed in the *chli1/fdx3* mutant, confirmed the successful rescue of the wild-type phenotype and the key role of CHLI1 in chlorophyll biosynthesis.

HPLC analysis of samples of the dark-incubated mutant and the wild-type strain revealed 145-fold higher levels of Proto in *chli1/fdx3*, while in the rescued strains accumulation of this tetrapyrrole decreased to 60% higher Proto levels compared with the wild type (Table 1). Chl *a* and *b* reached 3–35% and 10–50% of the wild-type level, respectively (Table 1).

### Homology between the CHLI proteins from selected organisms

The presence of two CHLI isoforms in *A. thaliana* and *C. reinhardtii* prompted a wider scale analysis of the homology between CHLIs from different organisms, to examine their occurrence and any phylogenetic correlation between their possible functions and their evolutionary origin.

The analyzed CHLI homologs were grouped into seven major distinct branches (I–VII; Fig. 1). Group I includes CHLI1 (At4g18480.1, genome TAIR10) of *A. thaliana*, which shows high similarity to one of the isoforms of *Arabidopsis lyrata* (946346, genome v1.0) and to other representatives of the Brassicaceae family (Fig. 1).

It is worth mentioning that all of the examined plants clustering in group I seem to have a second CHLI isoform, which is gathered in group II (Fig. 1), together with *B. rapa*, which seems to have even a third CHLI within this group (Brara.B02858.1). Based on the complementation of CHLI1 by CHLI2 in *A. thaliana* (Huang and Li, 2009), it would be interesting to compare the expression of CHLIs gathered in groups I and II, and their ability for reciprocal complementation.

Group III contains two CHLIs (Lus10015302 and Lus10025423, genome v1.0) from *Linum usitatissimum* (Linaceae family), which differ from each other only in seven amino acid residues (Fig. 1; Supplementary Fig. S9). It is likely that both sequences constitute the same protein and the difference originates from alternative sequencing or indeed a whole-genome duplication event (Wang *et al.*, 2012). However, the *Nicotiana tabacum* CHLI (NCBI: O22436.1) also belongs to this group, and its function in MgCh activity has been examined previously (Papenbrock *et al.*, 2000*b*).

Group IV includes CHLI1 of *C. reinhardtii* (Cre06.g306300.t1.2, genome v5.5), which shows high amino acid sequence similarity to Vocar20008053m (genome v2.0) of *Volvox carteri* (Fig. 1). Both organisms belong to the same order of Chlamydomonadales (Volvocales) of the class Chlorophyceae (green algae). Considering the evolutionary routes of the analyzed organisms, the presence of the *Synechocystis* sp. PCC6803 CHLI (WP_011243252.1) in group IV is not surprising, but such a high similarity might be indicative of a relatively conserved sequence of *C. reinhardtii* CHLI1. Still within group IV, two other CHLI isoforms of *Physcomitrella patens* (genome v3.0; Fig. 1) branch further away. However, locus Phpat.010G011100 seems to have two alternative transcripts, but the putative protein encoded by Phpat.010G011100.2 branches furthest away from all the analyzed putative CHLIs (Fig. 1). Therefore, it is likely that it might be assigned incorrectly. In the same line, the early release of *P. patens* genome v3.1 indicates only the Phpat.010G011100.2 transcript from this locus.

Group V contains CHLI2 (Cre12.g510800.t1.2) of *C. reinhardtii*, which shows high sequence similarity to the putative second isoform of CHLI from *V. carteri* (Vocar20000924m; Fig. 1). The third CHLI in this group originates from *Coccomyxa subellipsoidea* C-169 (35407, genome v2.0), which belongs to the same class of Chlorophyceae, but to the order of Chlorococcales. Although the CHLI domain is recognizable in these proteins (PFAM, PF01078; EC 6.6.1.1; KEGGORTH, K03405), the entire group branches further away from groups I, II, and III, and functions of these proteins other than in the MgCh complex cannot be excluded.

Putative CHLIs from *Micromonas* sp. (CCMP1545 and RCC299, genome v3.0) and *Ostreococcus lucimarinus* (29195, genome v2.0), which belong to the same family of Mamiellaceae, diverged from the same branch as group V, and were categorized separately in group VI. The only representative of group VII is *Rhodobacter sphaeroides* (NCBI: AAB97156.1), whose bacterial I subunit (BchI) seems to be the most distantly related to all of the CHLIs analyzed here.

Proteins from *Micromonas pusilla* CCMP1545 (22176), *Micromonas* sp. RCC299 (105016), *O. lucimarinus* (29195), *C. reinhardtii* (Cre12.g510800.t1.2), *V. carteri* (Vocar20 000924m), and *C. subellipsoidea C-169* (35407) possess a C-terminal 46–53 amino acid extension and shorter N-termini compared with all the other analyzed CHLI-like proteins, except CHLI from *Synechocystis* sp. and BchI from *R. sphaeroides* (Supplementary Fig. S9).

Analysis by TargetP 1.1 (http://www.cbs.dtu.dk/services/TargetP/, accessed 15 January 2016; Emanuelsson *et al.*, 2007) indicated that all the proteins from groups I–IV, except *Synechocystis* sp. PCC6803, carry the N-terminal sequence of a chloroplast transit peptide (cTP). The C-terminal 46–53 amino acid extensions of the proteins from group V and VI have been examined using the InterProScan 5 (http://www.ebi.ac.uk/Tools/pfa/iprscan5/, accessed 15 January 2016), but no indication with regard to the family membership, domains, repeats, or biological function could be determined.

### Overexpression of CHLI2 in *C. reinhardtii* has no effect on chlorophyll synthesis

The comparative analysis of the expression kinetics of *CHLI1* and *CHLI2* upon the shift from dark to light (Supplementary Fig. S3L) demonstrated lower expression of *CHLI2* compared with *CHLI1*, which resembles expression of the two *CHLI* genes in *A. thaliana* (Brenner *et al.*, 2000; Matsumoto *et al.*, 2004). Surprisingly, even CHLI2 expression did not contribute to low levels of chlorophyll, and *chli1/fdx3* showed a chlorophyll-free brown phenotype, which resembles the *chlh* and *chld* mutants. Therefore, the *C. reinhardtii* strains overexpressing CHLI2 in the *chli1/fdx3* background were generated and examined for chlorophyll and TBS intermediate accumulation, and light sensitivity.

**Fig. 3.**
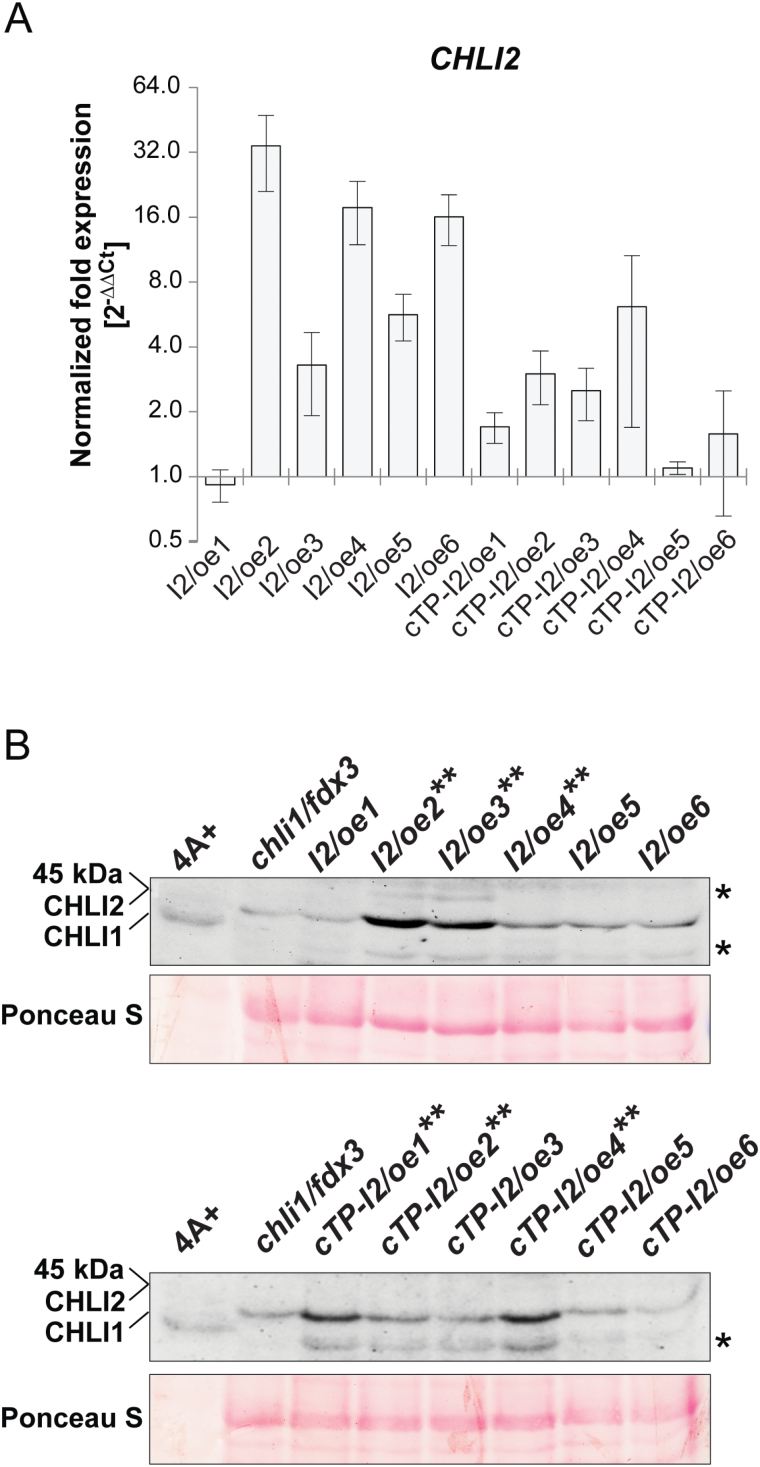
Analysis of CHLI2 overexpression in six strains obtained from the transformation with pCHLI2/GenD2 and six strains from the transformation with pCHLI2/GenD2-cTP. (A) Transcript levels of *CHLI2* analyzed by qRT–PCR; results represent the calculated fold expression (2^−ΔΔCt^) normalized to *chli1/fdx3*. Analyses were performed in biological triplicates, and error bars represent the SD. (B) Western blot showing the 40kDa CHLI1 and 45kDa CHLI2 protein levels in strains overexpressing CHLI2, compared with wild- type 4A+ and *chli1/fdx3*, respectively; the unspecific immunodetection of the protein with a molecular mass >45kDa is marked by an asterisk; strains selected for further analysis are marked with two asterisks; the lower panel shows the blot stained with Ponceau S used as a loading control. (This figure is available in colour at *JXB* online.)

Analyses performed by PredSL (Petsalaki *et al.*, 2006), TargetP 1.1 (Emanuelsson *et al.*, 2007), and Predotar (Small *et al.*, 2004) all indicated the presence of the cTP in a 417 amino acid sequence of CHLI1, while predictions of 425 amino acids of CHLI2 indicated possible targeting to a secretory pathway or mitochondria, but not to the chloroplast. Therefore, a dual approach was undertaken to overexpress the *CHLI2*-coding sequence driven by the *PSAD* promoter in the *chli1/fdx3* mutant background. The *CHLI2* sequence was fused with the sequence encoding the PSAD transit peptide, while the construct carrying exclusively the native CHLI2 targeting sequence was used independently as the control (Supplementary Fig. S4B). Following transformation with the pCHLI2/GenD2 plasmid without the cTP of PSAD, six colonies were randomly selected and labeled *I2/oe1*, *I2/oe2*, *I2/oe3*, *I2/oe4*, *I2/oe5*, and *I2/oe6*. Similarly, six colonies were selected following transformation with pCHLI2/GenD2-cTP and labeled *cTP-I2/oe1*, *cTP-I2/oe2*, *cTP-I2/oe3*, *cTP-I2/oe4*, *cTP-I2/oe5*, and *cTP-I2/oe6.* All selected transformants were examined for the overexpression of *CHLI2*, based on the transcript and protein levels compared with *chli1/fdx3* (Fig. 3A, B), following pre-growth in the dark and exposure to 20 μmol photons m^−2^ s^−1^ light, for 4h. Most of the strains transformed either with pCHLI2/GenD2 or pCHLI2/GenD2-cTP showed higher levels of CHLI2 mRNA compared with *chli1/fdx3* (Fig. 3A). Based on the amino acid sequence and online tools (http://proteome.gs.washington.edu/cgi-bin/aa_calc.pl, accessed 15 January 2016, and http://bioinformatics.org/sms/prot_mw.html, accessed 15 January 2016), the calculated masses of the mature *C. reinhardtii* CHLI1 and CHLI2 proteins are 40kDa and 45kDa, respectively. The 40kDa CHLI1 is absent in *chli1/fdx3* and all of the CHLI2-overexpressing strains, but it is present in the wild type (Fig. 3B). The CHLI2-overexpressor lines accumulate a protein of ~45kDa, which corresponds to the expected molecular mass of the mature CHLI2. Strains with the highest CHLI2 accumulation were selected for further analyses.

**Fig. 4.**
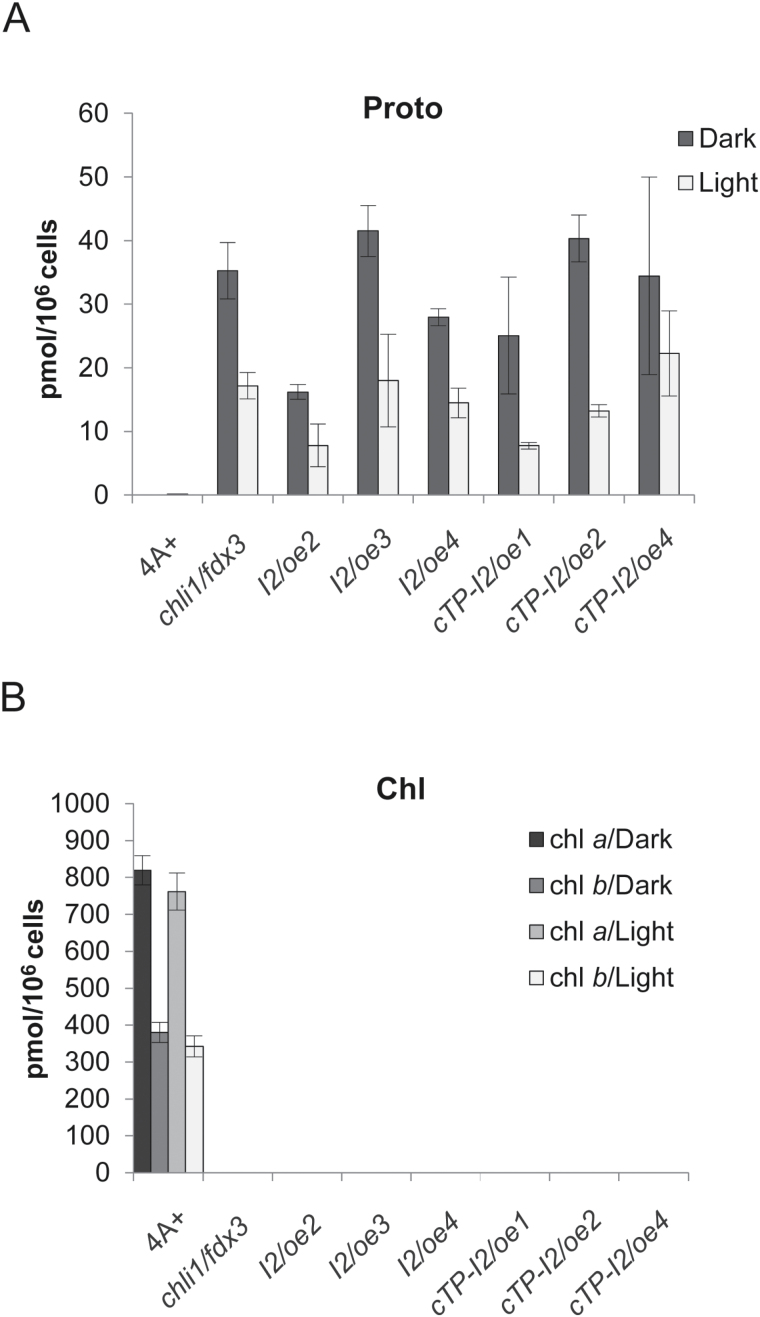
Analysis of the steady-state levels of Proto and chlorophyll in strains overexpressing CHLI2 with or without the cTP of PSAD in the dark and with 20 μmol photons m^−2^ s^−1^ compared with *chli1/fdx3* and the wild type. The experiment was performed in biological triplicates; error bars represent the calculated SD. (A) Proto; (B) Chl *a* and Chl *b* content.

Analysis of the Proto content and pigments also did not show any differences between *chli1/fdx3* and CHLI2-overexpressing strains. In all the strains, higher levels of Proto were observed compared with the wild type, both in the dark and upon 4h exposure to 20 µmol photons m^−2^ s^−1^, although certain variations in accumulation of this tetrapyrrole metabolite were observed (Fig. 4A). No PChlide, Chlide (Supplementary Fig. S6A, B), or even trace amounts of chlorophyll (Fig. 4B) could be detected in *chli1/fdx3* and CHLI2-overexpressors, as compared with the wild type. Accumulation of Proto seems to be affecting heme biosynthesis, resulting in higher levels of this tetrapyrrole (Supplementary Fig. S2C; von Gromoff *et al.*, 2008; Brzezowski *et al.*, 2014). However, no differences in heme content correlating with CHLI2 levels in comparison with *chli1/fdx3* were detected (Supplementary Fig. S6C). Additionally, carotenoid levels were determined in the dark and light, again showing no changes, which could be correlated with the CHLI2 levels (Supplementary Fig. S6D–I).

Examination of the light sensitivity in strains overexpressing CHLI2, regardless of the presence or absence of the PSAD chloroplast transit peptide, did not reveal any changes in *chli1/fdx3* phenotype. While the growth of the wild type positively correlated with the light intensities, *chli1/fdx3* and all the strains overexpressing CHLI2 were able to survive dark and light conditions in the examined range up to 30–40 µmol photons m^−2^ s^−1^, but 50 µmol photons m^−2^ s^−1^ light was always lethal.

### Silencing of *CHLI2* does not affect TBS or carotenoid biosynthesis

**Fig. 5.**
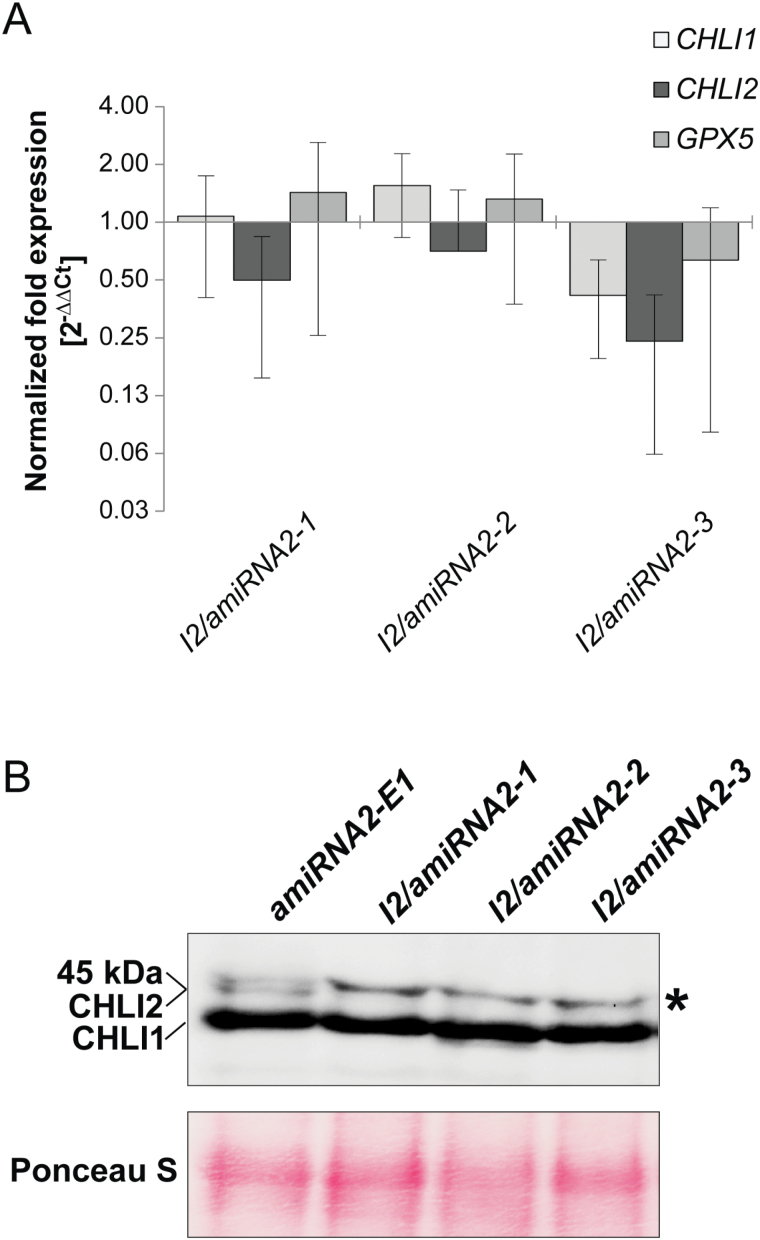
Analysis of the strains carrying CHLI2/amiRNA-E9/3'UTR plasmid for silencing of *CHLI2*. (A) Transcript levels of *CHLI1*, *CHLI2*, and *GPX5* analyzed by qRT–PCR;the results represent calculated fold expression (2^−ΔΔCt^) normalized to *amiRNA2-E1* transformed with the pChlamiRNA2 plasmid without the 90-mer dsDNA for amiRNA. Analyses were performed in biological triplicates; error bars represent the SD; the results are presented on a log_2_ scale. (B) Western blot analysis demonstrating silencing of *CHLI2* in *I2/amiRNA2-1*, *I2/amiRNA2-2*, and *I2/amiRNA2-3* compared with *amiRNA2-E1.* CHLI1 is also visible, and unspecific immunodetection of the protein with molecular mass >45kDa is marked by an asterisk; the lower panel shows the blot stained with Ponceau S as a loading control. (This figure is available in colour at *JXB* online.)

Because CHLI2 overexpression did not complement CHLI1 deficiency in *chli1/fdx3*, *CHLI2* was silenced in the wild-type background to identify any possible function of CHLI2 specifically in the TBS pathway. The amiRNA silencing system was applied using the pChlamiRNA2 vector (Molnar *et al.*, 2009). Following transformation of the *CC-3395* strain, three colonies were selected and annotated as *I2/amiRNA2-1*, *I2/amiRNA2-2*, and *I2/amiRNA2-3*. Additional transformation of *cc-3395* was conducted with pChlamiRNA2 without the designed 90bp dsDNA. Such a control strain annotated as *amiRNA2-E1* was necessary mainly to eliminate the need for arginine supplementation, which otherwise would cause differences in the rate of metabolism. Examination of the *CHLI2* mRNA in *I2/amiRNA2-1*, *I2/amiRNA2-2*, and *I2/amiRNA2-3* showed a decrease to 50, 71, and 24% of the *amiRNA2-E1* level, respectively, without a direct correlation with expression of *CHLI1* and the *GPX5* gene, which were used as the controls (Fig. 5A).

Western blot analysis revealed that both the 40kDa and 45kDa proteins could be detected in *amiRNA2-E1*. The amount of CHLI1 did not differ in *I2/amiRNA2-1*, *I2/amiRNA2-2*, and *I2/amiRNA2-3* compared with *amiRNA2-E1*. However, the 45kDa band corresponding to CHLI2 could not be detected in *I2/amiRNA2-1*, *I2/amiRNA2-2*, and *I2/amiRNA2-3* (Fig. 5B), confirming the silencing event in these strains. As seen in Fig. 3B, a non-specific binding of the CHLI1 antibody was always detected (Fig. 5B). Analysis of the potential light sensitivity and growth pattern in 50, 100, 200, and 300 µmol photons m^−2^ s^−1^ light did not reveal differences between the strains with silenced CHLI2 and *amiRNA2-E1* used as a control.

**Fig. 6.**
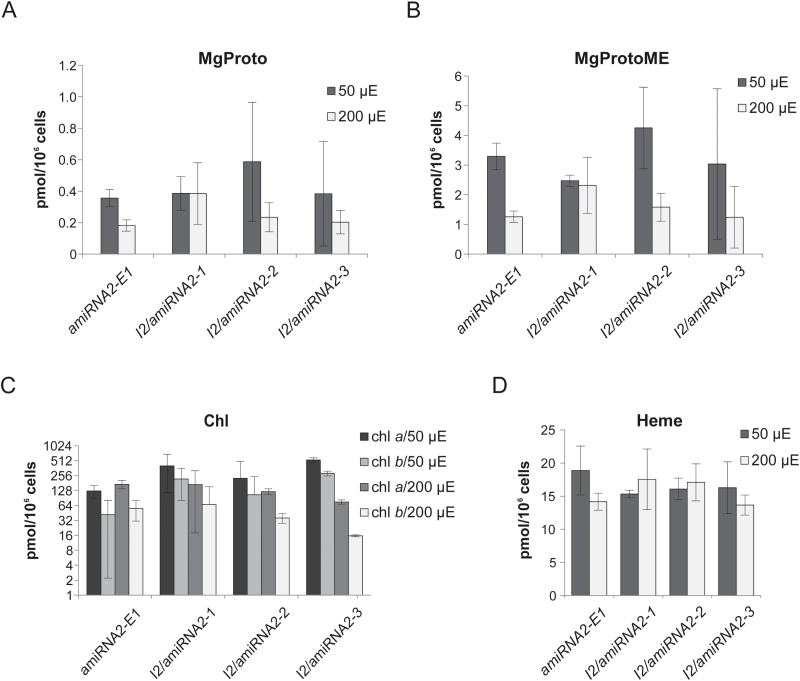
Analysis of the steady-state levels of selected TBS intermediates and end-products in *I2/amiRNA2-1*, *I2/amiRNA2-2*, and *I2/amiRNA2-3* in comparison with *amiRNA2-E1*, following exposure to 50 μmol photons m^−2^ s^−1^ and 200 μmol photons m^−2^ s^−1^ light. Analyses were performed in biological triplicate; error bars represent the SD. (A) MgProto, (B) MgProtoME, (C) chlorophyll, with results presented on a log_2_ scale, and (D) heme.

The steady-state levels of TBS pathway intermediates and end-products were analyzed in strains with silenced *CHLI2* and compared with the levels observed in *amiRNA2-E1* in 50 µmol photons m^−2^ s^−1^ and 200 µmol photons m^−2^ s^−1^ light. While Proto could not be detected under these light conditions, no correlation between MgCh activity and the reduced presence of CHLI2 could be determined, although a certain variation in MgProto levels was observed between biological replicates and strains (Fig. 6A). Also, accumulating MgProtoME, Chl *a*, and Chl *b* contents were not modified in *I2/amiRNA2-1*, *I2/amiRNA2-1* and *I2/amiRNA2-1* compared with *amiRNA2-E1* (Fig. 6B, C). Additionally, PChlide, Chlide, and carotenoid levels did not differ in relation to the CHLI2 deficiency at either 50 µmol photons m^−2^ s^−1^ or 200 µmol photons m^−2^ s^−1^ (Supplementary Fig. S7A–H).

Because Proto is a substrate for MgCh and FeCh, it was likely that any variations in Proto levels would affect not only the chlorophyll but also the heme biosynthesis in strains with silenced CHLI2, if it was involved in MgCh activity (Supplementary Figs S2C, S6C). However, the absence of CHLI2 did not result in different heme contents in *I2/amiRNA2-1*, *I2/amiRNA2-1*, and *I2/amiRNA2-1* compared with *amiRNA2-E1* (Fig. 6D).

## Discussion

### CHLI1 is indispensable for MgCh activity

Transformation of the *chli1/fdx3* mutant with a genomic version of *CHLI1* restored the wild-type phenotype (Fig. 2B; Table 1), pointing to a crucial role for this protein in MgCh activity. Although several genes neighboring *CHLI1* were also affected by the insertion of the DNA during the mutagenesis (Supplementary Fig. S1), introduction of the wild-type *CHLI1* was sufficient for the rescue of chlorophyll biosynthesis and recovery of the ability to grow photoautotrophically. In contrast, transformation with *FDX3* had no effect on these phenotypes, confirming that deletion of *CHLI1* is responsible for the defect in MgCh activity. Additionally, although FDX3 has been annotated as belonging to the FDX family in *C. reinhardtii*, it shows a high divergence compared with the other proteins from this group, and it clusters with FDXs with unknown function (Terauchi *et al.*, 2009). More recently it was suggested that FDX3 of *C. reinhardtii* might be involved in nitrogen assimilation (Peden *et al.*, 2013). Thus, it is unlikely that the mutation in *FDX3* would have a direct effect on tetrapyrrole biosynthesis.

### Lack of MgCh function due to the *chli1* knockout results in modulated chloroplast retrograde signaling

Disturbance in chloroplast functions results in reduced expression of PhAN genes. Mutations affecting TBS in *A. thaliana* were reported to cause impairments in the chloroplast retrograde signaling in *gun* mutants upon NF treatment. Despite the dysfunction in tetrapyrrole biosynthesis, the chloroplast did not show severely reduced transcripts for PhAN genes in the presence of NF compared with the wild-type chloroplasts (Susek *et al.*, 1993; Mochizuki *et al.*, 2001; Cottage *et al.*, 2007; Woodson *et al.*, 2011). In the same line, inactivation of CHLI and CHLH expression by antisense RNA synthesis in tobacco caused modified nuclear gene expression of PhAN and TBS genes, as a result of impaired chlorophyll synthesis and consequently reduced assembly of photosynthetic complexes (Papenbrock et al., 2000*a*, *b*). Different lines with disturbed retrograde signaling have been presented, such as mutants carrying defects in the CHLH subunit of MgCh, for example *gun5* of *A. thaliana* (Mochizuki *et al.*, 2001), or *brs-1* and *chl1* of *C. reinhardtii* (Kropat *et al.*, 1997; Chekunova *et al.*, 2001). It was also reported that the *xantha-f*, *-g*, and *-h* mutants of *Hordeum vulgare* L., with defects in *CHLH*, *CHLD*, and *CHLI* genes, respectively, are compromised in the normal plastid-derived control of nuclear gene expression (Gadjieva *et al.*, 2005). The GUN4 protein was also shown to play a role in chloroplast retrograde signaling in *A. thaliana* (Susek *et al.*, 1993; Mochizuki *et al.*, 2001; Larkin *et al.*, 2003) and *C. reinhardtii* (Formighieri *et al.*, 2012; Brzezowski *et al.*, 2014).

It is relevant to refer to the current discussion on plastid-derived signaling, which can be summarized in two controversial conceptual ideas of tetrapyrrole-mediated signaling. Retrograde signaling is either induced in response to adverse environmental conditions and compromised chloroplast biogenesis, or it is a part of a continuous exchange of information between plastids and the nucleus to balance metabolic activities (Pogson *et al.*, 2008).

In this line, it is worth mentioning that *cs* and *ch42* mutants of *A. thaliana*, carrying defects in CHLI, do not show the NF-dependent *gun* phenotype (Mochizuki *et al.*, 2001), but may compromise MgCh activity and chlorophyll supply, which both will hamper chloroplast function. In contrast, it was reported that the semi-dominant *chli1* (*cs215*/*cs215*) mutant and the *chli1*/*chli1 chli2*/*chli2* double mutant display a *gun* phenotype, because they accumulate a higher level of *LHCB1* transcript upon NF treatment than the wild type (Huang and Li, 2009). Because the *chli1* knockout mutant of *A. thaliana* does not display the *gun* phenotype, it was suggested that perhaps CHLI2 is sufficient for the signaling function in *chli1* of *A. thaliana* (Nott *et al.*, 2006). Analysis of the gene expression in *chli1/fdx3* conducted in the present study demonstrated a severe effect of the *CHLI1* knockout on PhAN and TBS genes, as the result of a plastid-mediated down-regulation (Supplementary Fig. S3). It is obvious that deficiency in chlorophyll synthesis is instantaneously communicated to alter nuclear gene expression, but in the light of the controversies on the contribution of metabolic products, it is worth emphasizing that in *chli1* a direct participation of neither one of the other MgCh subunits nor of Proto and MgProto in signaling transfer to the nucleus can be deduced from these studies. This also seems reasonable when inability to carry out of photosynthesis and ROS-mediated signaling also down-regulate expression of TBS and PhAN genes.

### The lack of the chlorophyll in the *chli1* knockout affects carotenoid composition

The accumulation of Proto and the lack of chlorophyll cause a general disturbance in cell metabolism and also affect the carotenoid levels, both in the dark and in light (Supplementary Fig. S2A, B).

Compared with the wild type, an accumulation of zeaxanthin and lower levels of violaxanthin, antheraxanthin, and β-carotene were observed in the dark and in light in *chli1/fdx3* (Supplementary Fig. S2A), while no change in the lutein content was detected either in dark or in light conditions (Supplementary S2A, B). The direct mechanism linking a defect in TBS with an alteration of carotenoid biosynthesis is unknown, and its elucidation would require more in-depth analysis. However, an interesting possibility is that the increased synthesis of heme in *chli1/fdx3* perturbs the Fe- and heme-dependent enzymes involved in epoxidation of zeaxanthin and hydroxylation of β-carotene. Perturbed zeaxanthin epoxidase is a possible cause of zeaxanthin accumulation in *chli1/fdx3* also because of the lower levels of antheraxanthin, violaxanthin, and neoxanthin in this strain compared with the wild type in the dark and after 4h exposure to light (Supplementary Figs S2A, S6E, F, H).

Zeaxanthin epoxidase uses reduced ferredoxin to epoxidize zeaxanthin (Bouvier *et al.*, 1996), but no difference in zeaxanthin levels could be observed between strains transformed with CHLI1/pMS188 or with both CHLI1/pMS188 and FDX3/pMS586 (data not shown), which reduces the possibility that the second site mutation, *fdx3*, could be responsible for the zeaxanthin accumulation phenotype. However, involvement of FDX3 cannot be excluded, because these strains do not accumulate high levels of Proto observed in *chli1/fdx3* (Table 1), and thus the *fdx3* mutation effect may not be apparent.

Zeaxanthin is an efficient ^1^O_2_ quencher, and it was demonstrated that the increased levels of this pigment may reduce damage to membrane lipids caused by ^1^O_2_ (Havaux *et al.*, 2007; Johnson *et al.*, 2007). Increased levels of zeaxanthin may not be surprising in high light conditions in the wild type, but are puzzling in *chli1/fdx3*, considering a photosynthesizing effect of accumulating Proto and most probably increased generation of ^1^O_2_ in this strain in light (Supplementary Fig. S2B). Increased zeaxanthin is also observed in the dark (Supplementary Fig. S2A). Nevertheless, because the role of xanthophyll cycle pigments is mainly correlated with the energy dissipation at photosystem II (PSII; Demmig-Adams, 1990; Aro *et al.*, 1993; Adams *et al.*, 1995; Niyogi *et al.*, 1998), a potential functionality of the xanthophyll cycle and accumulation of zeaxanthin in *chli1/fdx3* remains to be elucidated. Indeed, zeaxanthin formation was demonstrated to be involved in a mechanism other than non-photochemical quenching (NPQ; Havaux and Niyogi, 1999). Additionally, xanthophylls have been demonstrated to function independently from binding to PSII antenna in the *ch1* mutant of *A. thaliana* (Havaux *et al.*, 2007). The *ch1* mutant lacks Chl *b*, due to the null mutation in the gene encoding Chl *a* oxygenase, and does not assemble the PSII antenna complexes (Espineda *et al.*, 1999). It shows a low capacity for NPQ (Havaux *et al.*, 2007) and higher light sensitivity than the wild type (Havaux *et al.*, 2004). However, the *chli1/fdx3* mutant is not expected to assemble the functional photosystems at all, because of the complete lack of chlorophyll. This would be indicative of the other mechanism(s) governing the xanthophyll cycle, which are completely independent from the photosynthetic apparatus.

### CHLI2 is not involved in MgCh activity and does not affect chlorophyll biosynthesis

In the *chli1* knockout mutant of *A. thaliana*, chlorophyll is reduced to 10–17% of the wild-type level (Rissler *et al.*, 2002). The *C. reinhardtii chlh* and *chld* mutants show complete lack of chlorophyll (Chekunova *et al.*, 2001; von Gromoff *et al.*, 2008), just like the *chli1* knockout (Fig. 2; Supplementary Fig. S2A, B). Although the second isoform of the CHLH subunit also exists in *C. reinhardtii*, its function remains unknown. Previous experiments with the *chlh* mutant alleles indicated that the second *C. reinhardtii* CHLH cannot complement CHLH1 deficiency. In either case, results obtained in the present study indicate that the CHLI1 of *C. reinhardtii* might be the only isoform playing a role in MgCh formation.

CHLI2 of *C. reinhardtii* was overexpressed in the *chli1/fdx3* background, to examine whether higher levels of CHLI2 can functionally complement the *chli1* null mutation. Although higher levels of CHLI2 were observed in strains overexpressing the putative second CHLI isoform (Fig. 3), no correlation could be demonstrated between protein levels and MgCh function, or chlorophyll accumulation. Moreover, because of the presence of the light-independent protochlorophyllide reductase (DPOR) in *C. reinhardtii* and the ability to produce chlorophyll in the dark, the analyses were performed in both dark and light conditions, to examine the possibility of CHLI2 playing a role in any of these conditions. Nevertheless, based on the analysis of the TBS intermediates and end-products, no function in tetrapyrrole biosynthesis specifically could be assigned to CHLI2. However, it has to be mentioned that localization of the native CHLI2 should be examined in experiments involving cell fractionation, or localization studies involving green fluorescent protein (GFP)–CHLI2 or yellow fluorescent protein (YFP)–CHLI2 fusion proteins.

The potential functions of the CHLI2 protein were also examined in the *CC-3395* strain with the silenced *CHLI2* gene. The tetrapyrrole intermediates and end-products were analyzed in 50 µmol photons m^−2^ s^−1^ and 200 µmol photons m^−2^ s^−1^, but no difference could be observed between *I2/amiRNA2-1*, *I2/amiRNA2-2*, or *I2/amiRNA2-3* and the *amiRNA2-E1* strain used as the control. In the same line, it is not expected that the lower CHLI2 transcript levels correlate with modified nuclear gene expression for TBS and PhAN genes. Also, no difference could be determined between the strains with silenced *CHLI2* and the control strain from examination of the general appearance and light sensitivity at 50, 100, 200, or 300 µmol photons m^−2^ s^−1^ light conditions. In summary, all the results combined suggest that Mg chelation does not depend on CHLI2 as a MgCh subunit, and that the CHLI1 isoform in *C. reinhardtii* is the indispensable protein involved in the formation of the functional enzymatic complex.

### *In silico* analyses point to distinct roles for CHLI2 and CHLI1 in *C. reinhardtii*

Analysis of the 417 amino acid CHLI1 and 425 amino acid CHLI2 of *C. reinhardtii* using InterProScan 5 (http://www.ebi.ac.uk/Tools/pfa/iprscan5/, accessed 15 January 2016) and the NCBI database (http://www.ncbi.nlm.nih.gov, accessed 15 January 2016), based on the sequences obtained from Phytozome v10.2 (*C. reinhardtii* genome v5.5), showed that the same functional domains present in CHLI1 can also be identified in CHLI2, including a P-loop containing nucleoside triphosphate hydrolase (IPR027417) carrying an AAA+ ATPase domain (IPR003593), containing Walker A, Walker B, predicted phosphorylation sites, and the arginine finger motif (Supplementary Fig. S8A). However, the P-loop ATPase domain in CHLI2 seems to be incomplete, and its functionality as an ATPase has not been examined so far (Supplementary Fig. S8A). Alignments of the amino acid sequence using DNAMAN software indicated that CHLI2 shows only 48.7% similarity to CHLI1 in *C. reinhardtii* (Supplementary Fig. S8A). At present, no function can be assigned to CHLI2. Due to the low structural resemblance of both CHLI isoforms in *C. reinhardtii*, CHLI2 could act as a ‘surrogate protein’. This hypothesis is consistent with a replacement model, where both CHLI isoforms are synthesized in different amounts and the potential placeholder CHLI2 functions to complete the hexameric ring structure, which mainly consists of CHLI1 units, but lowers ATPase activity in conditions with attenuated chlorophyll synthesis. CHLI2 might be deposited when efficient MgCh activity is required.

In contrast, CHLI1 and CHLI2 in *A. thaliana* show 83% similarity in amino acid sequence (Supplementary Fig. S8B). Based on phylogenetic analysis (Fig. 1), these paralogs in *A. thaliana* resulted from gene duplication in the Brassicaceae. Perhaps that is why the overexpression of the native CHLI2 of *A. thaliana* can substitute for the CHLI1 deficiency in this organism (Huang and Li, 2009), but the same inter-relationship could not be observed in *C. reinhardtii*. Based on the phylogenetic analysis and the presence of *A. thaliana* CHLI2 (At5g45930.1) in group II (Fig. 1), together with the clustering pattern in group I and II, it can be hypothesized that group II gathers proteins whose expression might be lower compared with the major CHLI isoforms (group I), but which, according to previous results, might be essential under certain circumstances; for example, stress conditions (Moseley *et al.*, 2000).

Based on the phenotypes of the *chli1* knockout and the wild-type phenotype in strains rescued with the genomic version of the *CHLI1* gene, it can be concluded that CHLI1 plays an essential role in MgCh activity, which cannot be substituted by CHLI2. The possible function of CHLI2 in tetrapyrrole biosynthesis has been examined by overexpressing the cDNA version of CHLI2 in the *chli1/fdx3* background and by silencing of *CHLI2* in the wild-type background. Based on the analysis of transcript levels, accumulation of TBS intermediates, TBS pathway end-products, carotenoid levels, light sensitivity, or general growth characteristics, no effect caused by higher accumulation of CHLI2 or absence of this protein could be observed.

## Supplementary data

Supplementary data are available at *JXB* online.

Figure S1. Transcript analyses of the *CHLI1*-neighboring genes in the *chli1/fdx3* mutant strain.

Figure S2. Analysis of the steady-state levels of intermediates and end-products of TBS, and carotenoids in *chli1/fdx3* compared with the wild type.

Figure S3. Kinetics of the expression of selected genes upon shift from dark to 20 μmol photons m^−2^ s^−1^, examined at 1, 2, 4, and 8h time points.

Figure S4. Vectors used for *C. reinhardtii* transformations.

Figure S5. Examination of growth and sensitivity of *chli1/fdx3* to light of 10, 50, and 100 μmol photons m^−2^ s^−1^ PAR.

Figure S6. Steady-state levels of TBS intermediates, heme, and pigments in strains overexpressing CHLI2 in the dark and after exposure to 20 µmol photons m^−2^ s^−1^ light for 4h, compared with the wild type and *chli1/fdx3*.

Figure S7. Steady-state levels of TBS intermediates and pigments in strains with silenced CHLI2 in 50 µmol photons m^−2^ s^−1^ and 200 µmol photons m^−2^ s^−1^ light, compared with the control strain *amiRNA2-E1*.

Figure S8. Alignment of the amino acid sequence of two CHLI isoforms.

Figure S9. Aligned amino acid sequences of the CHLI proteins used to construct the phylogenetic tree presented in Fig. 1.

Table S1. Results of crosses between CAL029C_08 (*chli1/fdx3*) and the wild type.

Table S2. Primers used in this study; underlined sequences indicate restriction sites.

Supplementary Data
