# Supplementary material for: Mg chelatase in chlorophyll synthesis and retrograde signaling in Chlamydomonas reinhardtii: CHLI2 cannot substitute for CHLI1
Source: J Exp Bot. 2016 Jan 25;67(13):3925–38. doi: 10.1093/jxb/erw004 (PMC4915523; doi:10.1093/jxb/erw004)
Supplement: Supplementary Data [file supp_67_13_3925__index.html]

Mg chelatase in chlorophyll synthesis and retrograde signaling in Chlamydomonas reinhardtii: CHLI2 cannot substitute for CHLI1 — Mg chelatase in chlorophyll synthesis and retrograde signaling in Chlamydomonas reinhardtii: CHLI2 cannot substitute for CHLI1 — Supplementary Data 

# Mg chelatase in chlorophyll synthesis and retrograde signaling in *Chlamydomonas reinhardtii*: CHLI2 cannot substitute for CHLI1

## Supplementary Data

Data files

- Supplementary\_tables\_S1\_S2\_figures\_S1\_S9.pdf - Supplementary Data
