# Supplementary material for: Mg chelatase in chlorophyll synthesis and retrograde signaling in Chlamydomonas reinhardtii: CHLI2 cannot substitute for CHLI1
Source: J Exp Bot. 2016 Jan 25;67(13):3925–38. doi: 10.1093/jxb/erw004 (PMC4915523; doi:10.1093/jxb/erw004)
Supplement: Supplementary Data [file supp_erw004_Supplementary_tables_S1_S2_figures_S1_S9.pdf]

Table S1. Results of crosses between CAL029C_08 (*chli1/fdx3*) and wild type.

Cross	Complete tetrads (PD:NPD:TT)	Total wild-type progeny	Total mutant progeny	Progeny recombinant for paromomycin marker
CAL029C_08 *chli1 mt+* X *mt-*	6:0:0	39	34	0

Table S2. Primers used in this study; underlined sequences indicate restriction sites.

Target	Primer	Primer sequence (5' to 3')	Amplicon length (bp)
**rescue of the *chli1/fdx3***			
genomic *CHLI1;*	PB207	GGTACCCGGGCAGAACTTGCTGATAA	4282
Cre06.g306300	PB208	TTATAAGTTGGAAGGAGCGGCGCTAG	
genomic *FDX3;*	PB209	TTATAATCCGATGCAGATCCGATTTC	1556
Cre06.g306350	PB210	CACGTAGTGCTAACCTCATCTCGCCCCAG	
**RT-PCR and qRT-PCR**			
18S rRNA	PB67	ATGGCCGTTCTTAGTTGGTG	131
	PB68	TGGCTAAACGCCAATAGTCC	
18S rRNA[1]	PB69	GATGGCTACCACATCCAAGGAA	94
	PB70	AAGCGCCCGGTATTGTTATTTATT	
Cre06.g306200.t1.1	PB140	CACAATAAACCTGGACGTGCAG	105
	PB141	ACCGCAATGAGGATGATCTCG	
Cre06.g306250.t1.1	PB136	GAAACCCATTATGGCGATCGG	100
	PB137	CCCAACATTTGGTGGTAGCTG	
Cre06.g306250.t1.1	PB138	ATGACCGTAACGCTGCGTAC	100
	PB139	CGTGAACGACAGTGTTTAGCG	
FDX3; *Cre06.g306350.t1.2*	PB132	AAGGGCATCAGCTACAAGGTC	90
	PB133	GGCATCGAGGATGTATTGGTTG	
Cre06.g306400.t1.2	PB134	CTGGAGCGCACCTTTATGAAG	94
	PB135	AGTGGAACAGGTTCTCGATGAC	
GTR; *Cre07.g342150.t1.2*	PB187	CATCTGGAGAGCAGCAAGAAG	114
	PB188	TGCGTTGTGAATGGTGAGAC	
GSAT; *Cre03.g158000.t1.2*	PB189	ACCTGGAGAAGGTGACCAAG	115
	PB190	TTGCAGAAGAAGAAGCCAAAC	

Continues on the next page

Target	Primer	Primer sequence (5' to 3')	Amplicon length (bp)
UROS; *Cre09.g409100.t1.2*	PB63	ACCTGCAACCGTCCCTTTAG	105
	PB64	GTACACCGACACGTGACACC	
PPX1; *Cre09.g396300.t1.2*	PB191	AGAGTGTGGAGCAGTTCATCC	142
	PB192	TCCAGAATCCAGATCCTGTTG	
CHLM; *Cre12.g498550.t1.1*	PB61	GCTATGGCTTCTGAGATTGCTC	148
	PB62	CGTCGAATGACTTCATCTGC	
CRD1; *Cre07.g346050.t2.1*	PB43	GCCGCATCTTCATTGAGTTC	129
	PB44	ACATGAGCAGGAACATCTCG	
CTH1; *Cre12.g510050.t1.2*	PB45	AAGGTTGCTTCCGATGAGAC	138
	PB46	CTTGAACTCGTTCAGCATCG	
POR; *Cre01.g015350.t1.1*	PB102	ATCTCTGACCCCAAGCTGAAC	134
	PB103	TTGGCAGAGATGTCCCACAG	
DVR1; *Cre01.g042800.t1.2*	PB213	CCGGTGACATCACCTATTCC	78
	PB214	TCTTCACGATGTCGATCTGG	
CAO1; *Cre01.g043350.t1.2*	PB197	AAGCAGCATGGAGATTGAGG	131
	PB198	TCTCATCACGGAACATCACC	
CHLH1; *Cre07.g325500.t1.1*	PB53	ACATCAACGCCAAGAACTCC	99
	PB54	CATCCTCCTTGAGCATATCG	
CHLH2; *Cre11.g477625.t1.1*	PB130	TAACATGAGCGGCATCTTCC	105
	PB131	TTCATGTCATCTGGCTCGTC	
CHLI1; *Cre06.g306300.t1.2*	PB57	GCTGATTCTGAACGTGATCG	85
	PB58	ACGAATGGTGGTGGACTTG	
CHLI2; *Cre12.g510800.t1.2*	PB59	ATTTGTGGACTCGTGCAAGG	122
	PB60	AGCAAATGTCCGAGATCAGG	
CHLD; *Cre05.g242000.t1.2*	PB51	TACGTGGACGAGATCAACCTG	140
	PB52	TTGTAGGTGGCAATCAGCAG	

Continues on the next page

Target	Primer	Primer sequence (5' to 3')	Amplicon length (bp)
GUN4; *Cre05.g246800.t1.2*	PB49<br>PB50	AGCGCAACTGGGTTTACTTC<br>AGCCGAACTTGTTGTTGCTG	102
FeC; *Cre07.g339750.t1.2*	PB65<br>PB66	ACACGGATGAGTCCATCAAG<br>TCCTCCAACGTCTCAATGTG	100
LHCA3;<br>*Cre11.g467573.t1.1*	PB221<br>PB222	CACCAACATCAAGTGGTTCG<br>GAACTGCATAGCCACGATCTC	112
LHCBM1;<br>*Cre01.g066917.t1.1*	PB215<br>PB216	CGGACAGCTTCTGGTATGG<br>GAACTCGCCAGTCAGGTAGC	86
CP26; *Cre16.g673650.t1.1*	PB217<br>PB218	GAGATCCCCGAGTACCTGAAC<br>CTCGTTCTCGCGGTACTTG	127
CP29; *Cre17.g720250.t1.2*	PB219<br>PB220	GTTGACGAGAACGACCAGAAC<br>TCTCCGAGCTAACCTCATCG	91
GPX5; *Cre10.g458450.t1.1*	PB165<br>PB166	AACCCTTTCACTCACATGCTGTCT<br>CGAGCGGCGACAGGAGTA	103
**overexpression of *CHLI2***			
CHLI2 CDS;<br>*Cre12.g510800.t1.2*	PB313<br>PB314	GCCGGCATGCAGAGTCTCCAGGGTCA<br>GAATTCTTACCGACGAGGGGGCAAGCCA	1290
CHLI2 CDS;<br>*Cre12.g510800.t1.2*	PB317<br>PB314	CATATGATGCAGAGTCTCCAGGGTCA<br>GAATTCTTACCGACGAGGGGGCAAGCCA	1290
**silencing of *CHLI2***			
Exon 9/3'UTR junction of *CHLI2*	PB449<br>amiFor	ctagtTCGCCGGTAAAAAAGGGTGGAtctcg<br>ctgatcggcaccatgggggtggtggtgatcagcgcta<br>TCCAGCCTTTTTTACCGGCGAg	N/A
	PB450<br>amiRev	ctagcTCGCCGGTAAAAAAGGCTGGAtagcg<br>ctgatcaccaccacccccatggtgccgatcagcgaga<br>TCCACCCTTTTTTACCGGCGAa	

[1](Fischer *et al.*, 2009)

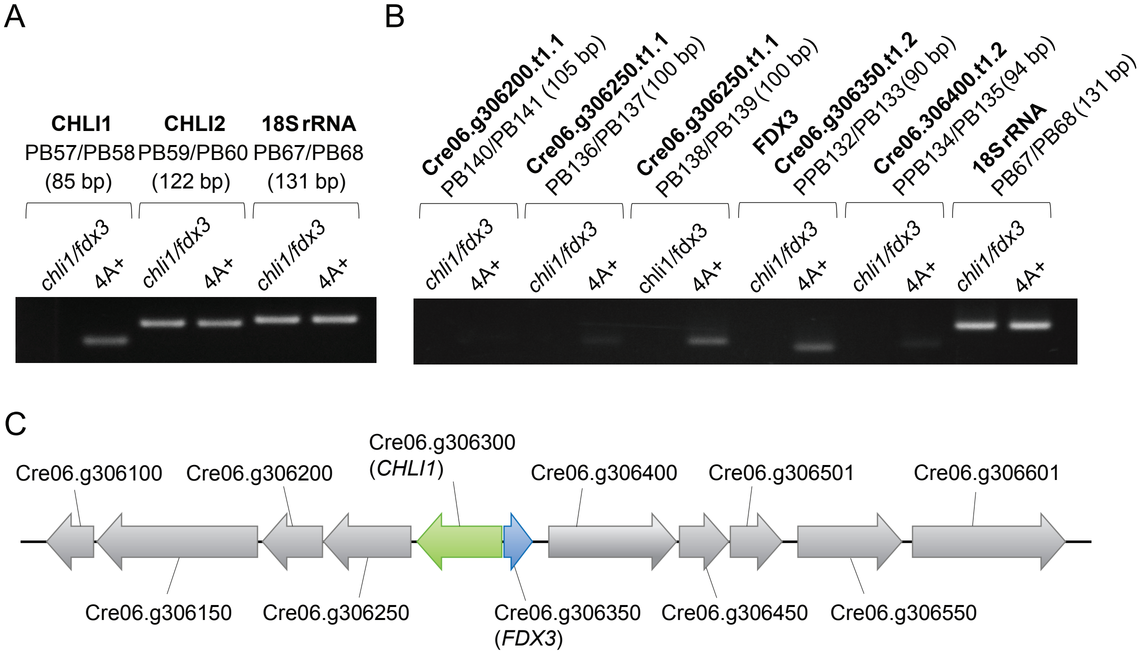

Figure S1. Transcript analyses of the *CHLI1* neighboring genes in the *chli1/fdx3* mutant strain. (A) RT-PCR analyses of the *CHLI1* and *CHLI2*. (B) *CHLI1* and the neighboring gene transcripts. Primers used for RT-PCR and the amplicon lengths are indicated in brackets. Primers used are listed in Table S2. (C) Map of a 51 kb DNA fragment of the chromosome 6, based on information obtained from the Phytozome database (*C. reinhardtii* genome v5.5, Phytozome v10.2, http://phytozome.jgi.doe.gov/pz/portal.html).

A

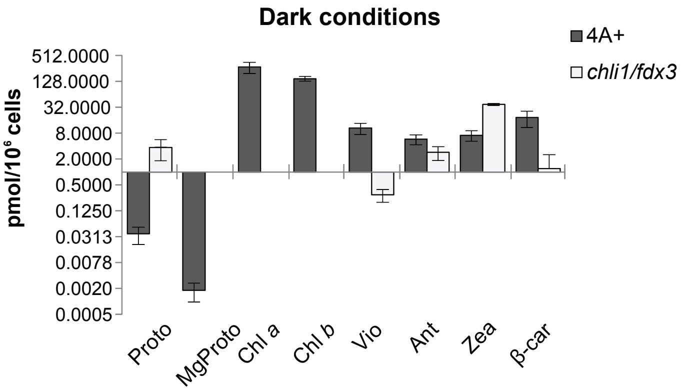

B

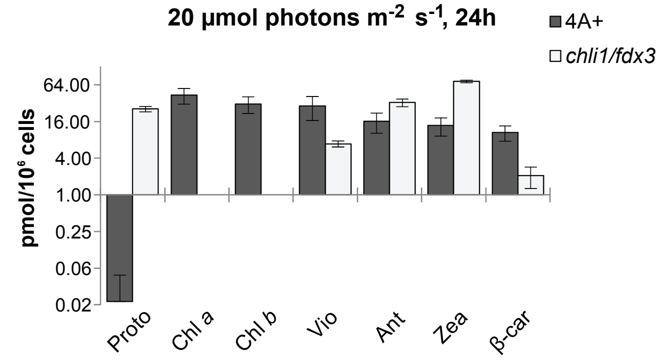

C

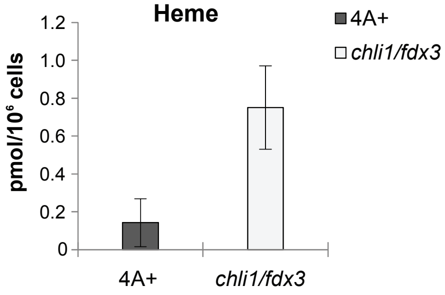

Figure S2. Analysis of the steady-state levels of intermediates and end-products of TBS, and carotenoids in *chli1/fdx3* compared to wild type. The experiment was performed in biological triplicates; error bars represent calculated standard deviation. (A) Steady-state levels of Proto, MgProto, chlorophyll and carotenoids in the dark; the results are presented on a $\log_2$ scale. MgProto, chlorophyll *a* and *b* were not detected in *chli1/fdx3*. (B) Steady-state levels of Proto, chlorophyll and carotenoids in the following exposure to 20 µmol photons m^{-2} s^{-1}, for 24 h; the results are presented on a $\log_2$ scale. MgProto was not detected in *chli1/fdx3* and wild type; chlorophyll *a* and *b* were not detected in *chli1/fdx3*. (C) Heme levels after 24 h exposure to 20 µmol photons m^{-2} s^{-1}.

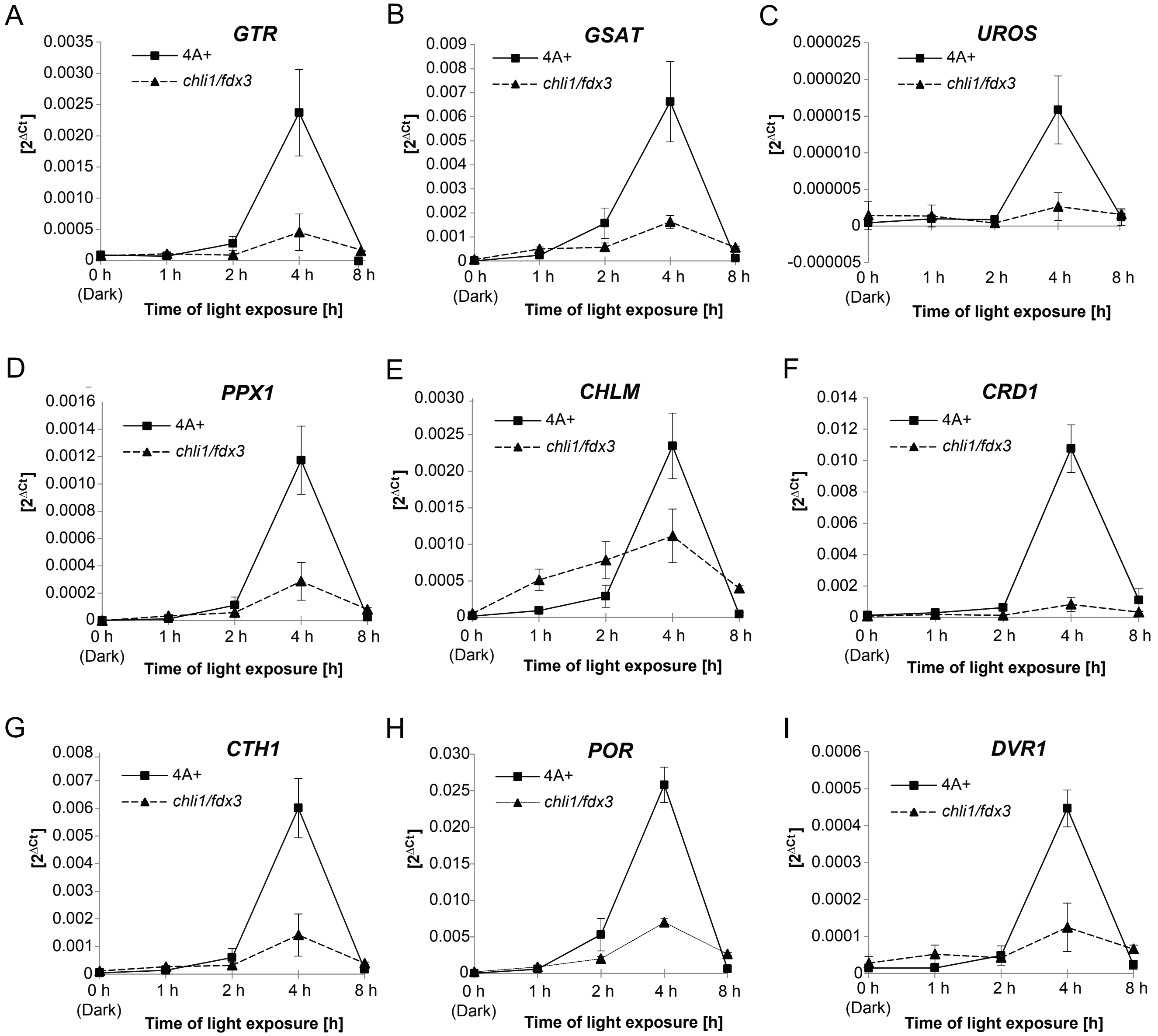

Figure S3. Kinetics of the expression of selected genes upon shift from dark to 20 µmol photons m^{-2} s^{-1}, examined at 1 h, 2 h, 4 h, and 8 h time-points. Presented transcript levels were calculated as $2^{\Delta Ct}$. Analyses were performed in biological triplicates; error bars represent calculated standard deviations. (A-O) Expression of the selected genes encoding enzymes of the TBS pathway; (A) glutamyl-tRNA reductase (*GTR*); (B) glutamate 1-semialdehyde aminotransferase (*GSAT*); (C) uroporphyrinogen III synthase (*UROS*); (D) protoporphyrinogen IX oxidase (*PPX1*); (E) Mg-protoporphyrin IX methyltransferase (*CHLM*); (F-G) two genes encoding subunits of Mg-protoporphyrin IX monomethylester cyclase (*CRD1* and *CTH1*); (H) light-dependent protochlorophyllide oxidoreductase (*POR*); (I) divinyl (proto)chlorophyllide vinyl reductase (*DVR1*); (J) chlorophyllide *a* oxidase (*CAO1*); (K) two isoforms encoding CHLH (*CHLH1* and *CHLH2*) subunit of MgCh, presented to compare expression of *CHLH1* and *CHLH2* in *chli1/fdx3* and wild type; (L) two isoforms encoding CHLI (*CHLI1* and *CHLI2*) subunit of MgCh, presented to compare expression of *CHLI1* and *CHLI2* in *chli1/fdx3* and wild type; (M) CHLD subunit of MgCh (*CHLD*); (N) *GENOMES UNCOUPLED 4* (*GUN4*); (O) ferrochelatase (*FeC*). (P-Q) Expression of genes encoding major light-harvesting proteins LHCA3 and LHCBM1; (R) minor light-harvesting, chlorophyll *a*/*b*-binding proteins LHCB5 (CP26).

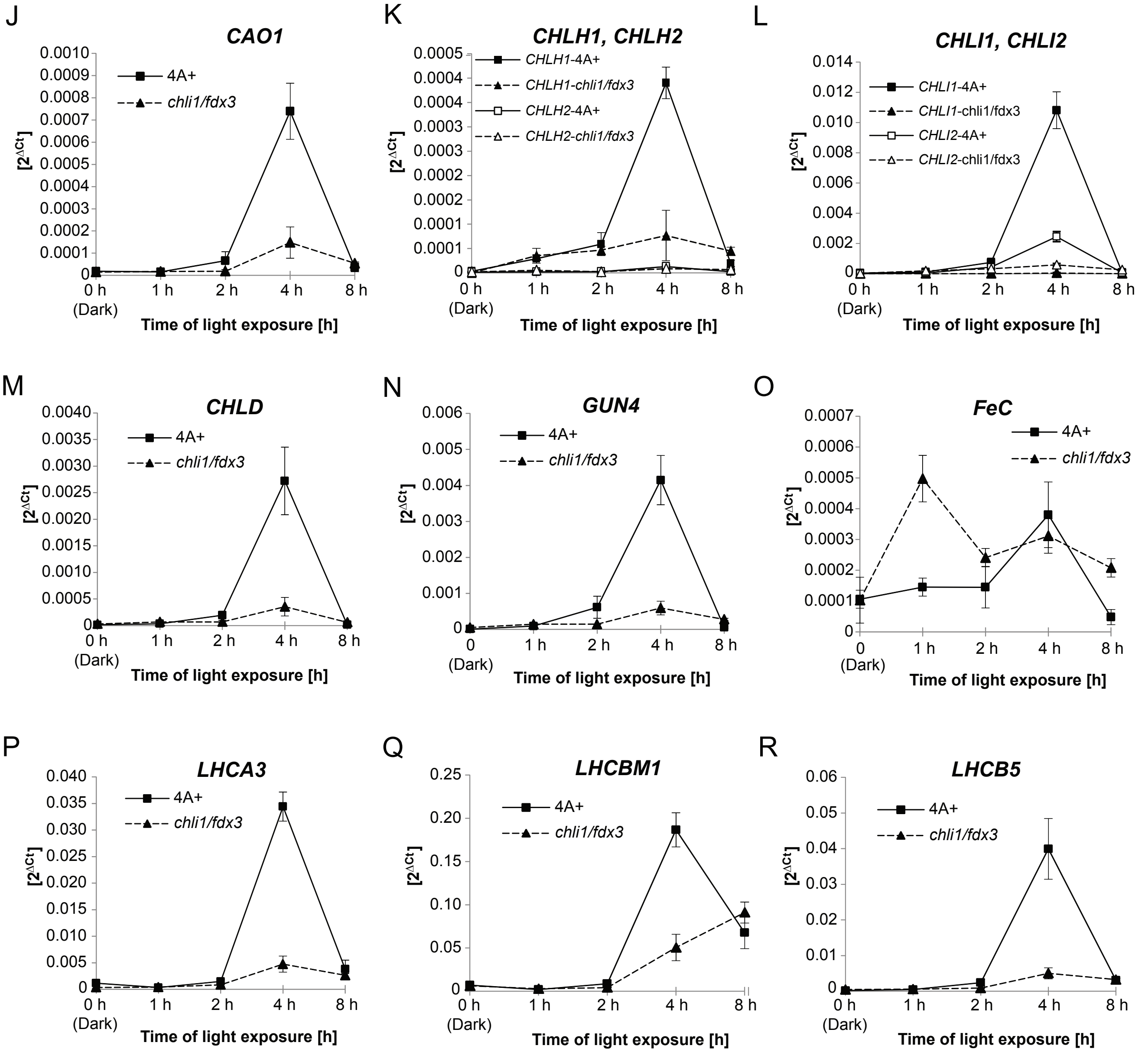

Figure S3. Kinetics of the expression of selected genes upon shift from dark to 20 µmol photons m^{-2} s^{-1}, examined at 1 h, 2 h, 4 h, and 8 h time-points. Presented transcript levels were calculated as $2^{\Delta Ct}$. Analyses were performed in biological triplicates; error bars represent calculated standard deviations. (A-O) Expression of the selected genes encoding enzymes of the TBS pathway; (A) glutamyl-tRNA reductase (*GTR*); (B) glutamate 1-semialdehyde aminotransferase (*GSAT*); (C) uroporphyrinogen III synthase (*UROS*); (D) protoporphyrinogen IX oxidase (*PPX1*); (E) Mg-protoporphyrin IX methyltransferase (*CHLM*); (F-G) two genes encoding subunits of Mg-protoporphyrin IX monomethylester cyclase (*CRD1* and *CTH1*); (H) light-dependent protochlorophyllide oxidoreductase (*POR*); (I) divinyl (proto)chlorophyllide vinyl reductase (*DVR1*); (J) chlorophyllide *a* oxidase (*CAO1*); (K) two isoforms encoding CHLH (*CHLH1* and *CHLH2*) subunit of MgCh, presented to compare expression of *CHLH1* and *CHLH2* in *chli1/fdx3* and wild type; (L) two isoforms encoding CHLI (*CHLI1* and *CHLI2*) subunit of MgCh, presented to compare expression of *CHLI1* and *CHLI2* in *chli1/fdx3* and wild type; (M) CHLD subunit of MgCh (*CHLD*); (N) *GENOMES UNCOUPLED 4* (*GUN4*); (O) ferrochelatase (*FeC*). (P-Q) Expression of genes encoding major light-harvesting proteins LHCA3 and LHCBM1; (R) minor light-harvesting, chlorophyll *a*/*b*-binding proteins LHCB5 (CP26).

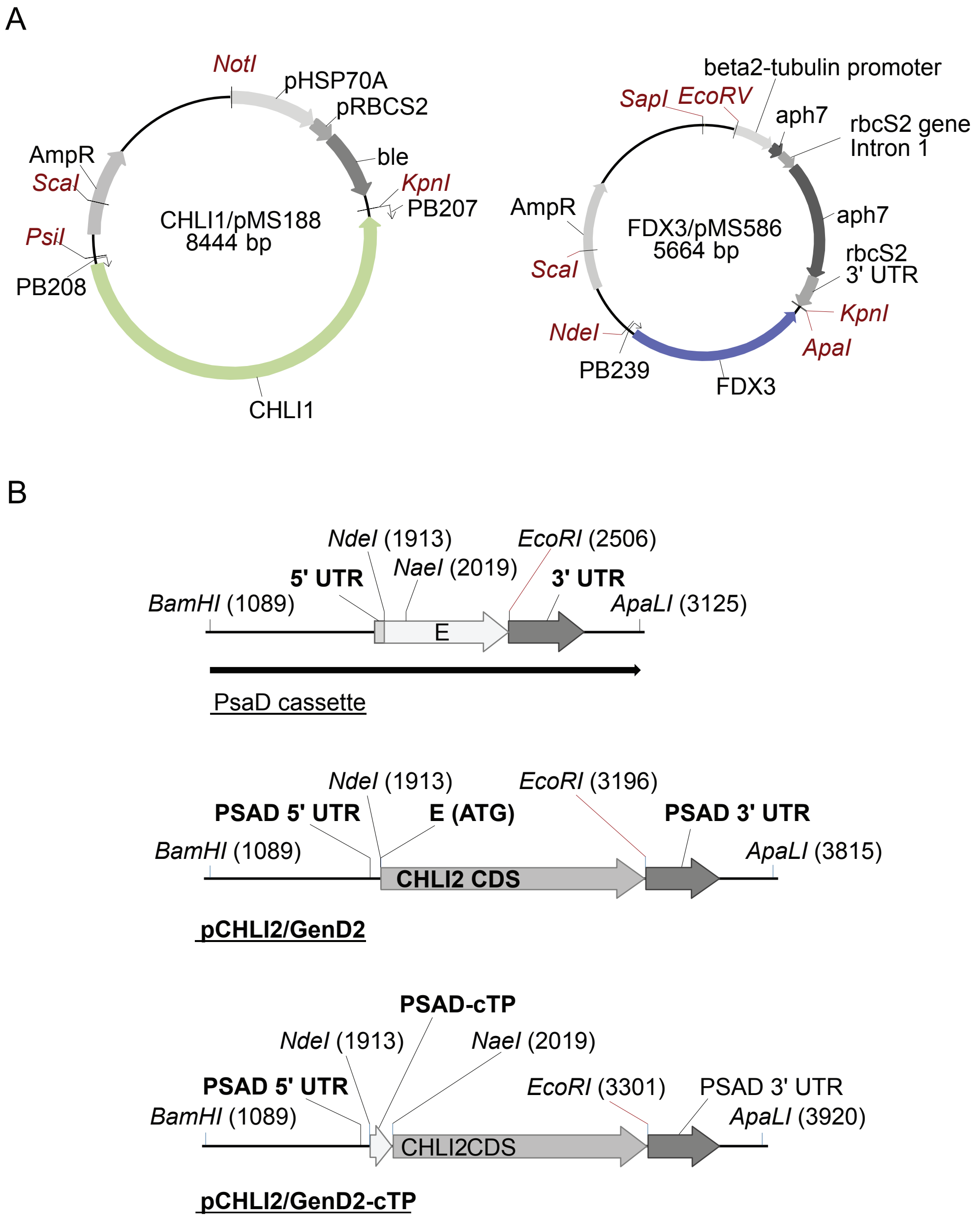

Figure S4. Vectors used for *C. reinhardtii* transformations. (A) The attempt to rescue the wild-type phenotype in the *chli1/fdx3* strain was conducted with CHLI1/pMS188 carrying genomic *CHLI1* and FDX3/pMS586 carrying genomic *FDX3*. (B) The PSAD cassette (Fischer and Rochaix, 2001) and vectors used for the overexpression of CHLI2 in *chli1/fdx3*, without or including the sequence for the PSAD chloroplast transit peptide (cTP), pCHLI2/GenD2 and pCHLI2/GenD2-cTP, respectively. The respective ligation sites are indicated.

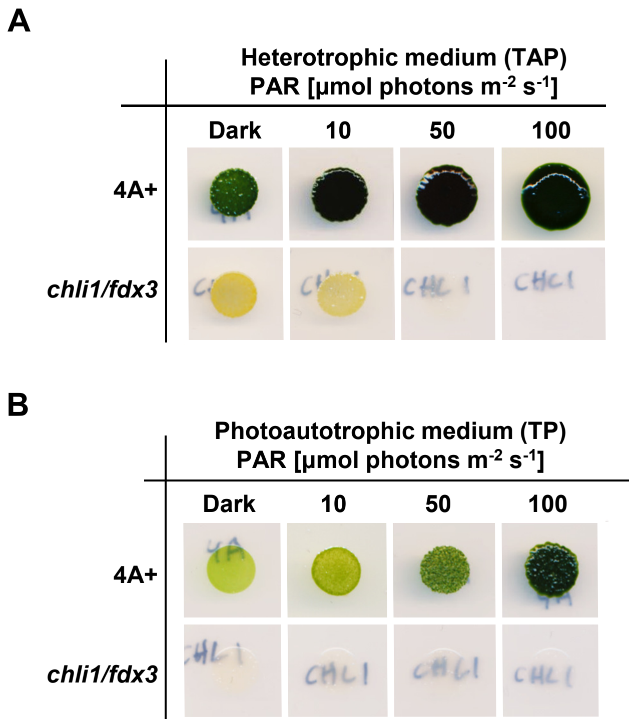

Figure S5. Examination of growth and sensitivity of *chli1/fdx3* to light of 10, 50, and 100 µmol photons m^{-2} s^{-1} PAR analyzed on (A) heterotrophic (TAP) and (B) photoautotrophic (Tris-phosphate, TP) media without acetate. The exposure of *chli1/fdx3* to ≥ 50 µmol photons m^{-2} s^{-1} was lethal, and the mutant was not able to grow photoautotrophically under any of the examined light conditions or in the dark.

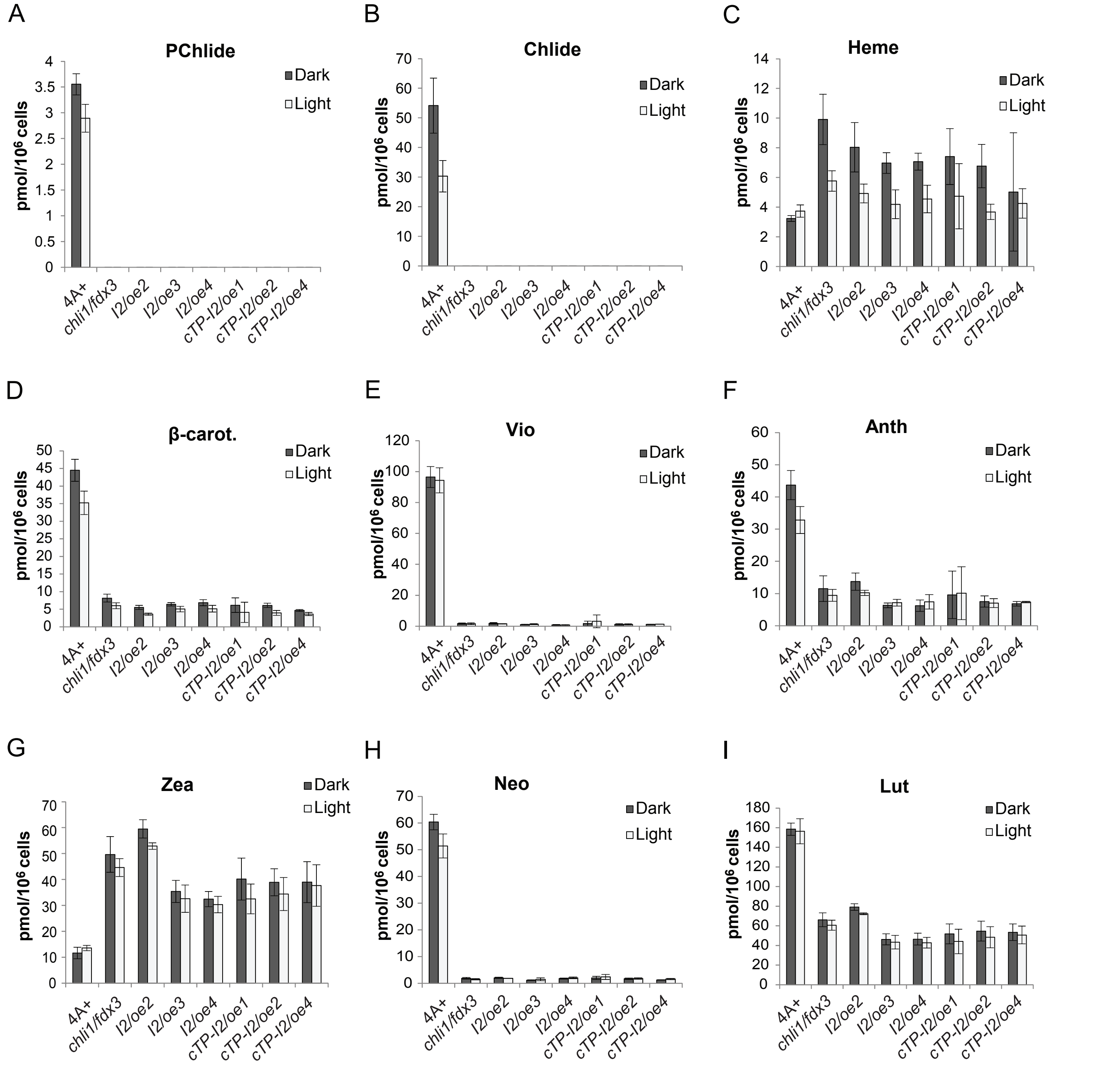

Figure S6. Steady-state levels of TBS intermediates, heme and pigments in strains overexpressing CHLI2 in dark and after exposure to 20 µmol photons m^{-2} s^{-1} light for 4 h, compared to wild type and *chli1/fdx3*. Analyses were performed in biological triplicates; error bars represent standard deviations. (A-B) substrate and product of POR, (A) Pchlide and (B) Chlide, respectively. (C) Heme levels; (D-I) carotenoids composition.

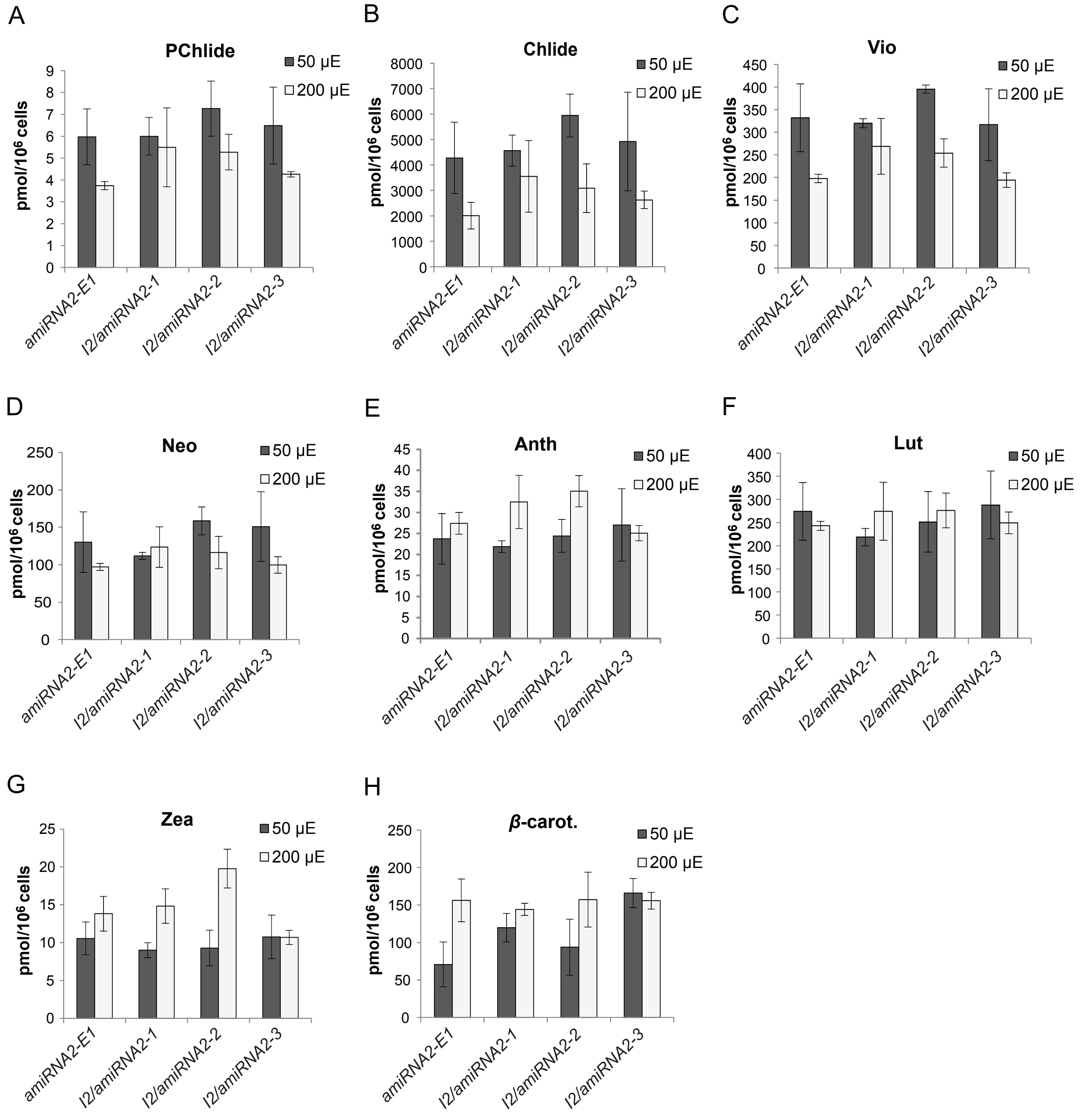

Figure S7. Steady-state levels of TBS intermediates and pigments in strains with silenced CHLI2 in 50 and 200 µmol photons m^{-2} s^{-1} light, compared to the control strain *amiRNA2-E1*. Analyses were performed in biological triplicates; error bars represent standard deviations. (A-B) substrate and product of POR, (A) Pchlide and (B) Chlide, respectively; (C-H) carotenoids composition.

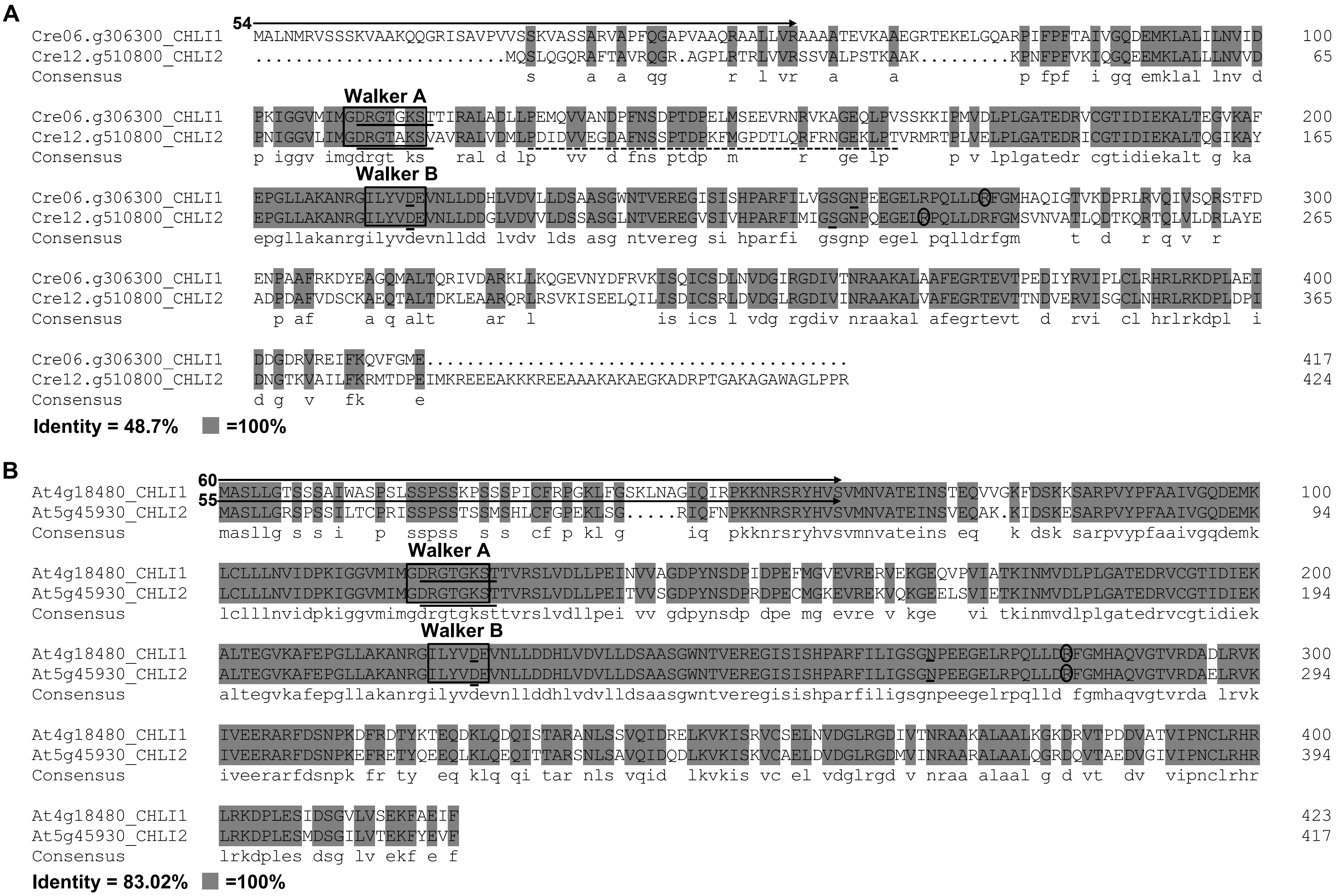
A
54
Walker A
Walker B
Cre06.g306300_CHLI1 MALNMRVSSSKVAAKQQGRISAVPVVSSKVASSARVAPFQGAPVAAQRAALLVRAAAATEVKAAEGRTEKELGQARPIFPFTAIVGQDEMKLALILNVID 100
Cre12.g510800_CHLI2 .........................MQSLQGQRAFTAVRQGR.AGPLRTRLVVRSSVALPSTKAAK.........KPNFPFVKIQGQEEMKLALLLNVVD 65
Consensus s a a qg r l vr a a p fpf i gq emklal lnv d
Cre06.g306300_CHLI1 PKIGGVMIMGDRGTGKSTTIRALADLLPEMQVVANDPFNSDPTDPELMSEEVRNRVKAGEQLPVSSKKIPMVDLPLGATEDRVCGTIDIEKALTEGVKAF 200
Cre12.g510800_CHLI2 PNIGGVLIMGDRGTAKSVAVRALVDMLPDIDVVEGDAFNSSPTDPKFMGPDTLQRFRNGEKLPTVRMRTPLVELPLGATEDRICGTIDIEKALTQGIKAY 165
Consensus p iggv imgdrgt ks ral d lp vv d fns ptdp m r ge lp p v lplgatedr cgtidiekalt g ka
Cre06.g306300_CHLI1 EPGLLAKANRGILYVDEVNLLDDHLVDVLLDSAASGWNTVEREGISISHPARFILVGSGNPEEGELRPQLLDRFGMHAQIGTVKDPRLRVQIVSQRSTFD 300
Cre12.g510800_CHLI2 EPGLLAKANRGILYVDEVNLLDDGLVDVVLDSSASGLNTVEREGVSIVHPARFIMIGSGNPQEGELRPQLLDRFGMSVNVATLQDTKQRTQLVLDRLAYE 265
Consensus epgllakanrgilyvdevnlldd lvdv lds asg ntvereg si hparfi gsgnp egel pqlldrfgm t d r q v r
Cre06.g306300_CHLI1 ENPAAFRKDYEAGQMALTQRIVDARKLLKQGEVNYDFRVKISQICSDLNVDGIRGDIVTNRAAKALAAFEGRTEVTPEDIYRVIPLCLRHRLRKDPLAEI 400
Cre12.g510800_CHLI2 ADPDAFVDSCKAEQTALTDKLEAARQRLRSVKISEELQILISDICSRLDVDGLRGDIVINRAAKALVAFEGRTEVTTNDVERVISGCLNHRLRKDPLDPI 365
Consensus p af a q alt ar l is ics l vdg rgdiv nraakal afegrtevt d rvi cl hrlrkdpl i
Cre06.g306300_CHLI1 DDGDRVREIFKQVFGME.................................... 417
Cre12.g510800_CHLI2 DNGTKVAILFKRMTDPEIMKREEEAKKKREEAAAKAKAEGKADRPTGAKAGAWAGLPPR 424
Consensus d g v fk e
Identity = 48.7% =100%
B
60
55
Walker A
Walker B
At4g18480_CHLI1 MASSLLGTSSSAIWASPSLSSPSSKPSSSPICFRPGKLFGSKLNAGIQIRPKKNRSRYHVSVMNVATEINSTEQVVGKFDSKKSARPVYPFAAIVGQDEMK 100
At5g45930_CHLI2 MASSLLGRSPSSILTCPRISSPSSTSSMSHLCFGPEKLSG.....RIQFNPKKNRSRYHVSVMNVATEINSVEQAK.KIDSKESARPVYPFAAIVGQDEMK 94
Consensus masllg s s i p sspss s s cf p kl g iq pkknrsryhvsvmnvateins eq k dsk sarpvypfaaivgqdemk
At4g18480_CHLI1 LCLLLNVIDPKIGGVMIMGDRGTGKSTTVRSLVDLLPEINVVAGDPYNSDPIDPEFMGVEVRERVEKGEQVPVIATKINMVDLPLGATEDRVCGTIDIEK 200
At5g45930_CHLI2 LCLLLNVIDPKIGGVMIMGDRGTGKSTTVRSLVDLLPEITVVSGDPYNSDPRDPECMGKEVREKVQKGEELSVIETKINMVDLPLGATEDRVCGTIDIEK 194
Consensus lclllnvidpkiggvmimgdrgtgksttvrslvdllpei vv gdpynsdp dpe mg evre v kge vi tkinmvdlplgatedrvcgtidiek
At4g18480_CHLI1 ALTEGVKAFEPGLLAKANRGILYVDEVNLLDDHLVDVLLDSAASGWNTVEREGISISHPARFILIGSGNPEEGELRPQLLDRFGMHAQVGTVRDADLRVK 300
At5g45930_CHLI2 ALTEGVKAFEPGLLAKANRGILYVDEVNLLDDHLVDVLLDSAASGWNTVEREGISISHPARFILIGSGNPEEGELRPQLLDRFGMHAQVGTVRDAELRVK 294
Consensus altegvkafepgllakanrgilyvdevnllddhlvdvlldsaasgwntveregisishparfiligsgnpeegelrpqllld fgmhaqvgtvrda lrvk
At4g18480_CHLI1 IVEERARFDSNPKDFRDTYKTEQDKLQDQISTARANLSSVQIDRELKVKISRVCSELNVDGLRGDIVTNRAAKALAALKGKDRVTPDDVATVIPNCLRHR 400
At5g45930_CHLI2 IVEERARFDSNPKEFRETYQEEQLKLQEQITTARSNLSAVQIDQDLKVKISKVCAELDVDGLRGDMVINRAARALAALQGRDQVTAEDVGIVIPNCLRHR 394
Consensus iveerarfdsnpk fr ty eq klq qi tar nls vqid lkvkis vc el vdglrgd v nraa alaal g d vt dv vipnclrhr
At4g18480_CHLI1 LRKDPLESIDSGVLVSEKFAEIF 423
At5g45930_CHLI2 LRKDPLESMDSGILVTEKFYEVF 417
Consensus lrkdples dsg lv ekf e f
Identity = 83.02% =100%

Figure S8. Alignment of amino acid sequence of two CHLI isoforms. The alignments were performed using DNAMAN program Version 6, based on sequences obtained from the *C. reinhardtii* genome v5.5 and *A. thaliana* genome TAIR10, available at the Phytozome platform (v10.2); loci and transcript names are indicated. Walker A and Walker B motifs are indicated by frame, the ATP binding sites are underlined, and arginine fingers are marked with ovals. (A) CHLI1 (Cre06.g306300) and CHLI2 Cre12.g510800) of *C. reinhardtii*, demonstrating 48.7% homology. The 54 amino acid chloroplast transit peptide of CHLI1 is indicated by the horizontal arrow; Based on the in silico analyses, the CHLI2 of *C. reinhardtii* is missing the chloroplast transit peptide, and the gap from position 93 to 129 in the P-loop ATPase domain in this protein is underlined with dashed line. (B) CHLI1 (At4g18480) and CHLI2 (At5g45930) of *A. thaliana*, demonstrating 83.02% homology. The 60 amino acid chloroplast transit peptide, present in CHLI1 as well as in CHLI2 is indicated.

```
CCM_22176                    ................................................   0
RCC_105016                   ................................................   0
Olu_29195                    ........................................MRCSSAPR   8
Cre_Cre12.g510800.t1.2       ................................................   0
Vca_Vocar20000924m           ................................................   0
Csu_35407                    ................................................   0
Aly_494386                   ....55 MASLLGASPS...SIFTC.PRLSSPSST...SSISLVCFG...................PG.   35
Ath_At5g45930.1              ....55 MASLLGRSPS...SILTC.PRISSPSST...SSMSHLCFG...................PE.   35
Cgr_Cagra.2097s0009.1        ....56 MASLLRASPS...TTVLTRPRLSSPSSS...SSISPVCFG...................PG.   36
Cru_Carubv10026479m          ....56 MASLLRASPS...TTVLTRPRLSSPSSS...SSISPVCFG...................PG.   36
Bst_Bostr.8819s0123.1        ....56 MASVLGTSLS...SILTCPPRLSSPSST...SSISPVCFG...................PG.   36
Esa_Thhalv10000877m          MLHFEMIPSLQRVKENLQSCISLSNYRDEFSKLEKLLESSLTMASLLGTSAS...TILTC.PRFSSPSST...TSVSPVCFG...................PGK   78
Bra_Brara.B02858.1           ....44 MVSLLGTSPS...SILSC.PRLSSIPLT...S...PVCFR...................PGN   33
Bra_Brara.K01115.1           ....63 MASLLGTSSS...AICAS.HSLSSSSST...PSISPICFK...................PGK   36
Esa_Thhalv10025291m          ....60 MASLLGTSSS...AIWAS.PSLSSSSST...PSLSPICFR...................PGK   36
Aly_946346                   ....60 MASLLGTSSS...AIWAS.PSLSSSSST...PSTSPICFR...................PGK   36
Ath_At4g18480.1              ....60 MASLLGTSSS...AIWAS.PSLSSPSSK...PSSSPICFR...................PGK   36
Cgr_Cagra.2935s0008.1        ....61 MASLLGTSSS...AIWAA.PSLSSSSSS..THSTSPICFR...................PGK   37
Cru_Carubv10004877m          ....62 MASLLGTSSS...AIWAA.PSLSSSSSSS.THSTATICFR...................PGK   38
Bst_Bostr.30275s0323.1       ....63 MASLLGTSSS...AIWAA.PSLSSSSSSSLTPSSSSICFR...................PGN   39
Bra_Brara.K00442.1           ....60 MASLLGTSS.....IRAS.PSLSSSSSSS.TPSISLICFR...................PGR   36
Lus_Lus10015302              ....68 MASILGNSS...AAILSSRPICSSTSKSS..SSLPSLSLLP...................SGQ   39
Lus_Lus10025423              ....75 MASILGNSS...AAILSSRPICSSTSKSS..SSFPSVSLLP...................SGQ   39
Ptr_Poptri.011G063400.1      ....25 MATILGTSS...AAILAYRPFSK........PSIPSLSLTS...................SGL   33
Mtr_Medtr4g087895.1          ....70 MASTLSSSS...IAVLSSRRHSS........PSIHSLSLT...................KGQ   32
Egr_Eucgr.D00551.1           ...... MAHTLQYQN...QKEARHRQPATRHATGEEEPAREALALRDEEAMASLLGTSSTAILASRRLSPLSVQ   65
Nt_O22436.1                  ....50 MASLLGTSSSAAAAILASTPLSSRSCK....PAVFSLFPS...................SGQ   39
Ppa_Phpat.010G011100.1       ....58 MALLARGAVAAVAAVGFSNTTSKQGHQNGG..SNGVLAFRG...................NAF   42
Ppa_Phpat.010G011100.2       ...... MMGWDFWTQSSSKGDARPVFPFTAIVGQEE..MKMCLILNV...................IDP   42
Ppa_Phpat.002G065900.1       ....55 MACLVREAAAAVAAVSASSSTTKQAHLNG...STGVLACRR...................SSF   41
Cre_Cre06.g306300.t1.2       ....55 MALNMRVSSS...KVAAKQQG..RISAVPVVSSKVASSAR......................   35
Vca_Vocar20008053m           ....56 MALNMRTVSS...NVVAQQQHGARTPVRVPVNAKSVVTLR......................   37
PCC6803_WP_010871795.1       ................................................   0
Rs_AAB97156.1                ................................................   0
Consensus                           masllg s s    i    p  ss s      s                          g
```

Continues on the next page

Figure S9. Aligned amino acid sequences of the CHLI proteins used to construct phylogenetic tree presented in Figure 1. The alignment was performed using DNAMAN program Version 6, based on sequences obtained from Phytozome v10.2 (http://phytozome.jgi.doe.gov/pz/portal.html and NCBI protein sequence database (http://www.ncbi.nlm.nih.gov/protein; protein names are indicated). The identity ≥ 50% is indicated as orange, ≥ 75% as blue, and 100% as a grey highlight. Only predicted (TargetP 1.1) chloroplast transit peptides (cTPs) are indicated by arrows, in each case the predicted cTP length is shown. Walker A and Walker B motifs are indicated by frame, the ATP binding sites are underlined, and arginine fingers are marked with ovals. The gap from position 93 to 129 in the P-loop ATPase domain in CHLI2 (transcript Cre12.g510800.t1.2) is underlined with dashed line The respective transcript names are preceded by the following coding for the species names: *Micromonas pusilla* (CCM), *Micromonas* sp. RCC299 (RCC), *Ostreococcus lucimarinus* (Olu), *Chlamydomonas reinhardtii* (Cre), *Volvox carteri* (Vca), *Coccomyxa subellipsoidea* (Csu), *Arabidopsis lyrata* (Aly), *Arabidopsis thaliana* (Ath), *Capsella grandiflora* (Cgr), *Capsella rubella* (Cru), *Boechera stricta* (Bst), *Eutrema salsugineum* (Esa), *Brassica rapa* (Bra), *Linum usitatissimum* (Lus), *Populus trichocarpa* (Ptr), *Medicago truncatula* (Mtr), *Eucalyptus grandis* (Egr), *Nicotiana tabacum* (Nt), *Physcomitrella patens* (Ppa), *Synechocystis* sp. PCC6803 (PCC6803), and *Rhodobacter sphaeroides* (Rs).

```
                                                                                                   Walker A
CCM_22176                   ......MAGTPLAARARASVAKTGKRASRGS.LVIRASAE....TDAAAALVERGYPFVKIVGQDELKLALTLNVVDSKIGGCLIMGDRGTAKSVAVRALSDLLPDIDIV   99
RCC_105016                  ...MSAMATIQLAGAPLVARRGSAKKASKANRRAMRCNAA....TAAEVEGKVISYPFVKLVGQEELKLALILNVIDSRIGGCLIMGDRGTGKSVAVRALSDLLPEIDVV  103
Olu_29195                   RAPGAARARTTRAVADKTRARATAATTVGGRARASDARRA....TVTRAKSESASFPFVKIVAQEELKLALTLNVVDSAIGGVLIMGDRGTAKSVSVRSLVQLLPEIDVV  114
Cre_Cre12.g510800.t1.2      ........MQSLQGQRAFTAVRQGRAGPLRTRLVVRSSVA....LPSTKAAKKPNFPFVKIQGQEEMKLALLLNVVDPNIGGVLIMGDRGTAKSVAVRALVDMLPDIDVV   98
Vca_Vocar20000924m          ................................................MSSKKPNFPFVKIQGQEEMKLALLLNVVDPNIGGVLIMGDRGTGKSVAVRALVDLLPLISVV   62
Csu_35407                   ....................................................MPSLPFVKVAEQEDMKLALMLNVIDPTIGGVLIMGERGTGKSVAVRAMVDLLPEIEVV   58
Aly_494386                  ....KICGRIQFNPKKNKSRYHVS.VMNVATEINYVEQGK....KFDSKESARPVYPFAAIVGQDEMKLCLLLNVIDPKIGGVMIMGDRGTGKSTTVRSLVDLLPEIMVV  136
Ath_At5g45930.1             ....KLSGRIQFNPKKNRSRYHVS.VMNVATEINSVEQAK....KIDSKESARPVYPFAAIVGQDEMKLCLLLNVIDPKIGGVMIMGDRGTGKSTTVRSLVDLLPEITVV  136
Cgr_Cagra.2097s0009.1       ....KFCGRIELKPKKNRTRYHVS.VMNVATEINSIEQEK....KFDSKESARPVYPFAAIVGQDEMKLCLLLNVIDPKIGGVMIMGDRGTGKSTTVRSLVDLLPEITVV  137
Cru_Carubv10026479m         ....KFCGRIELKPKKNRTRYHVS.VMNVATEINSIEQEK....KFDSKESARPVYPFAAIVGQDEMKLCLLLNVIDPKIGGVMIMGDRGTGKSTTVRSLVDLLPEITVV  137
Bst_Bostr.8819s0123.1       ....KIFGRIQLKPKKNRSRYHVS.VMNVATEINSVEHAK....KFDSKESARPVYPFAAIVGQDEMKLCLLLNVIDPKIGGVMIMGDRGTGKSTTVRSLVDLLPEITVV  137
Esa_Thhalv10000877m         ICGKKLCGRIQLEPKKNRSRYRVSVVMNVATEINSIEQAK....KIDSKESARPVYPFAAIVGQDEMKLCLLLNVIDPKIGGVMIMGDRGTGKSTTVRSLVDLLPEITVV  184
Bra_Brara.B02858.1          TFGGKLYRRIQSETKKSRSRHHVL.VTNVATGINSIEQAK....KIDTKESARPVYPFAAIVGQDEMKLCLLLNVIDPKIGGVMIMGDRGTGKSTTVRSLVDLLPEITVV  138
Bra_Brara.K01115.1          ICGGKLNAGIQIRPKKNRSRHHLS.VVNVATEINSTEQVG....KFDSKKSARPVYPFAAIVGQDEMKLCLLLNVIDPKIGGVMIMGDRGTGKSTTVRSLVDLLPEIKVV  141
Esa_Thhalv10025291m         ICGAKLNAGIQIRPKKNKSRRHVS.VMNVATEINSTEQVG....KFDSKKSARPVYPFAAIVGQDEMKLCLLLNVIDPKIGGVMIMGDRGTGKSTTVRSLVDLLPEIEVV  141
Aly_946346                  LFGSKLNAGIQIRPKKNRSPYHVS.VVNVATEINSTEQVG....KFDSKKSARPVYPFAAIVGQDEMKLCLLLNVIDPKIGGVMIMGDRGTGKSTTVRSLVDLLPEINVV  141
Ath_At4g18480.1             LFGSKLNAGIQIRPKKNRSRYHVS.VMNVATEINSTEQVV...GKFDSKKSARPVYPFAAIVGQDEMKLCLLLNVIDPKIGGVMIMGDRGTGKSTTVRSLVDLLPEINVV  142
Cgr_Cagra.2935s0008.1       ISGRNLNAGIQMRPKKNRSRYHVS.VMNVATEINSTEQVE....KFDSKKSARPVYPFAAIVGQDEMKLCLLLNVIDPKIGGVMIMGDRGTGKSTTVRSLVDLLPEIKVV  142
Cru_Carubv10004877m         ISGRNFNAGIQMRPKKNRSRYHVS.VMNVATEINSTEQVE....KFDSKKSARPVYPFAAIVGQDEMKLCLLLNVIDPKIGGVMIMGDRGTGKSTTVRSLVDLLPEIKVV  143
Bst_Bostr.30275s0323.1      IFGRKLNAGIQIRPKKNRSRYHVS.VMNVATEINSTEQVE....KFDSKKSARPVYPFAAIVGQDEMKLCLLLNVIDPKIGGVMIMGDRGTGKSTTVRSLVDLLPEIKVV  144
Bra_Brara.K00442.1          ICGRALNAGIQIRRKKNRSRHHVS.VMNVATEINSTEQQV...GKFDSKKSARPVYPFAAIVGQDEMKLCLLLNVIDPKIGGVMIMGDRGTGKSTTVRSLVGLLPEITVV  142
Lus_Lus10015302             SYWRKQYCNGGVK..KGRSQLHIAN...VATEINAVEP...AA.RVADKESQRPVYPFAAIVGQDEMKLCLLLNVIDPKIGGVMIMGDRGTGKSTTVRSLTDLLPEIRVV  140
Lus_Lus10025423             SYWRKQYCNGGVK..KGRSQLHIAN...VATEINAVEP...AA.RVADKESRRPVYPFAAIVGQDEMKLCLLLNVIDPKIGGVMIMGDRGTGKSTTVRSLTDLLPEIRVV  140
Ptr_Poptri.011G063400.1     SFGRESYGGIGLVGKKGRPQFHVAVAC.VATDIGSVEE...AQ.KLASKENQRPVYPFAAIVGQDEMKLCLLLNVIDPKIGGVMIMGDRGTGKSTTVRSLVDLLPEIKVV  138
Mtr_Medtr4g087895.1         VSGCKFCGGIGFHGVKGKSQFSVLS...VATEINATQQDIQAQ.RIAYKESQRPVYPFSAIVGQDEMKLCLLLNVIDPKIGGVMIMGDRGTGKSTTVRSLVDLLPEIKVV  138
Egr_Eucgr.D00551.1          NHGKKCYGGIGFQAKKGRSPWHLAVTN.VATDINSVEQ...AQ.KLASKESQRPVYPFAAIVGQDEMKLCLLLNVIDPKIGGVMIMGDRGTGKSTTVRSLVDLLPEIQVV  170
Nt_O22436.1                 SQGRKFYGGIRVPVKKGRSQFHVAISN.VATEINLLKN...RV.RNLLEESQRPVYPFAAIVGQDEMKLCLLLNVIDPKIGGVMIMGDRGTGKSTTVRSLVDLLPEIKVI  144
Ppa_Phpat.010G011100.1      CRAVAARTSSVGCQVRVKRGAGPLRVTNVATLEQET......SAESSSKGDARPVFPFTAIVGQEEMKMCLILNVIDPKIGGVMIMGDRGTGKSTTVRALVDLLPEIQVV  146
Ppa_Phpat.010G011100.2      KIGGVMIMGDRGTGKSTTVRALVDLLPEIQVVAGDP......FNSSPEDPELMSEEVRKRVQANESLPVTTSRINMVDLPLGATEDRVCGTIDIEKALTEGVKAFEPGLL  146
Ppa_Phpat.002G065900.1      CQGASSRVSWSRCRSGERRAGRALRINNVAIPVKEQENL...TDNAASEGEARPVFPFTAIVGQEEMKMCLILNVIDPKIGGVMIMGDRGTGKSTTVRALVDLLPEIEVV  148
Cre_Cre06.g306300.t1.2      ...VAPFQGAPVAAQRAALLVRAA....AATEVKAAEGRT.....EKELGQARPIFPFTAIVGQDEMKLALILNVIDPKIGGVMIMGDRGTGKSTTIRALADLLPEMQVV  133
Vca_Vocar20008053m          ...VAPFQGAAVVPQRAALQVRAA....AATEVKP..........EKELGQARPIFPFTAIVGQDEMKLALILNVIDPKIGGVMIMGDRGTGKSTTIRALADLLPEMKVV  130
PCC6803_WP_010871795.1      ....................MTAT....LAAPSKT................RRVVFPFTAIVGQDEMKLALLLNVIDPKIGGVMIMGDRGTGKSTTIRALADLLPEIEVV   70
Rs_AAB97156.1               ...................................................MKKPFPFSAIVGQEQMKQAMVLTAIDPGIGGVLVFGDRGTGKSTAVRALAALLPLIKAV   59
Consensus                       k    i    kk rs  hv    nvateins e       k  sk sarpvypfaaivgqdemklclllnvidpkiggvmimgdrgtgksttvrslvdllpei vv
```

Continues on the next page

Figure S9. Aligned amino acid sequences of the CHLI proteins used to construct phylogenetic tree presented in Figure 1. The alignment was performed using DNAMAN program Version 6, based on sequences obtained from Phytozome v10.2 (http://phytozome.jgi.doe.gov/pz/portal.html and NCBI protein sequence database (http://www.ncbi.nlm.nih.gov/protein; protein names are indicated). The identity ≥ 50% is indicated as orange, ≥ 75% as blue, and 100% as a grey highlight. Only predicted (TargetP 1.1) chloroplast transit peptides (cTPs) are indicated by arrows, in each case the predicted cTP length is shown. Walker A and Walker B motifs are indicated by frame, the ATP binding sites are underlined, and arginine fingers are marked with ovals. The gap from position 93 to 129 in the P-loop ATPase domain in CHLI2 (transcript Cre12.g510800.t1.2) is underlined with dashed line The respective transcript names are preceded by the following coding for the species names: *Micromonas pusilla* (CCM), *Micromonas* sp. RCC299 (RCC), *Ostreococcus lucimarinus* (Olu), *Chlamydomonas reinhardtii* (Cre), *Volvox carteri* (Vca), *Coccomyxa subellipsoidea* (Csu), *Arabidopsis lyrata* (Aly), *Arabidopsis thaliana* (Ath), *Capsella grandiflora* (Cgr), *Capsella rubella* (Cru), *Boechera stricta* (Bst), *Eutrema salsugineum* (Esa), *Brassica rapa* (Bra), *Linum usitatissimum* (Lus), *Populus trichocarpa* (Ptr), *Medicago truncatula* (Mtr), *Eucalyptus grandis* (Egr), *Nicotiana tabacum* (Nt), *Physcomitrella patens* (Ppa), *Synechocystis* sp. PCC6803 (PCC6803), and *Rhodobacter sphaeroides* (Rs).

```
                                                                                                                           Walker B
CCM_22176                  PGDPFNSSPTDPELMGPEVLEKFRAKEDIPTGAMKIPMVEVPLGTTEDRICGTIDIEKALAEGVKAYDPGLLARANRGLLYIDEVNLLDDSLVDVVLDSAAGGWNTVERE   209
RCC_105016                 EGDAFNSSPTDPQLMGPEALEAFKAGLELTWAKMKVPMVEVPLGTTEDRICGTIDIEKALAEGVKAYDAGLLARANRGLLYIDEVNLLDDSLVDVVLDSAAGGWNTVERE   213
Olu_29195                  KNDPFNSSPTNPELMGPDVREAFQRGETLETAKMRVPMVEVPLGTTEDRICGTIDIEKALSEGTKAYDPGLLAKANRGLLYIDEVNLLDDSLVDVVLDSAAGGWNTVERE   224
Cre_Cre12.g510800.t1.2     EGDAFNSSPTDPKFMGPDTLQRFRNGEKLPTVRMRTPLVELPLGATEDRICGTIDIEKALTQGIKAYEPGLLAKANRGILYVDEVNLLDDGLVDVVLDSSASGLNTVERE   208
Vca_Vocar20000924m         EGDPFNSSPTDPKVMGPDVLDRWQRGEKLPTTQIRTPLVELPLGATEDRICGTIDIEKALTQGVKAYEPGLLAKANRGILYVDEVNLLDDGLVDVVLDSSASGLNTVERE   172
Csu_35407                  AEDAFNSHPTDTKLMGPDVLQRHRNGEQLPMVRVKTPLVELPLGATEDRICGTINIEKALQEGVKAYEPGLLAKANRGILYVDEVNLLDDGLVDVVLDSSASGVNTVERE   168
Aly_494386                 AGDPYNSDPRDPECMGKEVREKVQKGEQLPVIETKINMVDLPLGATEDRVCGTIDIEKALTEGVKAFEPGLLAKANRGILYVDEVNLLDDHLVDVLLDSAASGWNTVERE   246
Ath_At5g45930.1            SGDPYNSDPRDPECMGKEVREKVQKGEELSVIETKINMVDLPLGATEDRVCGTIDIEKALTEGVKAFEPGLLAKANRGILYVDEVNLLDDHLVDVLLDSAASGWNTVERE   246
Cgr_Cagra.2097s0009.1      AGDPYNSDPRDPEFMGKEVREKVQKGEKLPVIETKINMVDLPLGATEDRVCGTIDIEKALTEGVKAFEPGLLAKANRGILYVDEVNLLDDHLVDVLLDSAASGWNTVERE   247
Cru_Carubv10026479m        AGDPYNSDPRDPEFMGKEVREKVQKGEQLPVIETKINMVDLPLGATEDRVCGTIDIEKALTEGVKAFEPGLLAKANRGILYVDEVNLLDDHLVDVLLDSAASGWNTVERE   247
Bst_Bostr.8819s0123.1      AGDPYNSDPRDPEFMGKEVRERAQKGEQLPVLETKINMVDLPLGATEDRVCGTIDIEKALTEGVKAFEPGLLAKANRGILYVDEVNLLDDHLVDVLLDSAASGWNTVERE   247
Esa_Thhalv10000877m        AGDPYNSDPRDPEFMGKEVREKVEKGEKLPVIETKINMVDLPLGATEDRVCGTIDIEKALTEGVKAFEPGLLAKANRGILYVDEVNLLDDHLVDVLLDSAASGWNTVERE   294
Bra_Brara.B02858.1         AGDPYNSDPRDPEFMGKEVRERVRKGEELDVMETKINMVDLPLGATEDRVCGTIDIEKALTEGVKAFEPGLLAKANRGILYVDEVNLLDDHLVDVLLDSAASGWNTVERE   248
Bra_Brara.K01115.1         AGDPYNSDPLDPEFMGVEVRERVERGEQVPVVATKINMVDLPLGATEDRVCGTIDIEKALTEGVKAFEPGLLAKANRGILYVDEVNLLDDHLVDVLLDSAASGWNTVERE   251
Esa_Thhalv10025291m        AGDPYNSDPLDPEFMGVEVRERVERGEQVPVVATKINMVDLPLGATEDRVCGTIDIEKALTEGVKAFEPGLLAKANRGILYVDEVNLLDDHLVDVLLDSAASGWNTVERE   251
Aly_946346                 AGDPYNSDPIDPEFMGVEVRERVEKGEKVPVIATKINMVDLPLGATEDRVCGTIDIEKALTEGVKAFEPGLLAKANRGILYVDEVNLLDDHLVDVLLDSAASGWNTVERE   251
Ath_At4g18480.1            AGDPYNSDPIDPEFMGVEVRERVEKGEQVPVIATKINMVDLPLGATEDRVCGTIDIEKALTEGVKAFEPGLLAKANRGILYVDEVNLLDDHLVDVLLDSAASGWNTVERE   252
Cgr_Cagra.2935s0008.1      AGDPYNSDPIDPEFMGVEVRERVEKGEKVPVIATKINMVDLPLGATEDRVCGTIDIEKALTEGVKAFEPGLLAKANRGILYVDEVNLLDDHLVDVLLDSAASGWNTVERE   252
Cru_Carubv10004877m        AGDPYNSDPIDPEFMGVEVRERVEKGEKVPVIATKINMVDLPLGATEDRVCGTIDIEKALTEGVKAFEPGLLAKANRGILYVDEVNLLDDHLVDVLLDSAASGWNTVERE   253
Bst_Bostr.30275s0323.1     AGDPYNSDPIDPEFMGVEVRERVERGEQVPVIATKINMVDLPLGATEDRVCGTIDIEKALTEGVKAFEPGLLAKANRGILYVDEVNLLDDHLVDVLLDSAASGWNTVERE   254
Bra_Brara.K00442.1         AGDPYNSDPLDPEFMGVEVRERVERGEQVPVIATKINMVDLPLGATEDRVCGTIDIEKALTEGVKAFEPGLLAKANRGILYVDEVNLLDDHLVDVLLDSAASGWNTVERE   252
Lus_Lus10015302            SGDPYNSDPEDPEAMGMEVRESVNKGKELTVVLTKINMVDLPLGATEDRVCGTIDIEKALTEGVKAFEPGLLAKANRGILYVDEVNLLDDHLVDVLLDSAASGWNTVERE   250
Lus_Lus10025423            SGDPYNSDPEDPEAMGMEVRESVNKGKELTVVLTKINMVDLPLGATEDRVCGTIDIEKALTEGVKAFEPGLLAKANRGILYVDEVNLLDDHLVDVLLDSAASGWNTVERE   250
Ptr_Poptri.011G063400.1    AGDPYNSDPEDPESMGIEVRESVVKGENLTVVMTKINMVDLPLGATEDRVCGTIDIEKALTEGVKAFEPGLLAKANRGILYVDEVNLLDDHLVDVLLDSAASGWNTVERE   248
Mtr_Medtr4g087895.1        AGDPYNSDPEDPEFMGIEVRERVLKGEQLEVVFSKINMVDLPLGATEDRVCGTIDIEKALTEGVKAFEPGLLAKANRGILYVDEVNLLDDHLVDVLLDSAASGWNTVERE   248
Egr_Eucgr.D00551.1         AGDPYNSDPQDPESMGVEVRELVIKGEQLPVVSTKINMVDLPLGATEDRVCGTIDIEKALTEGVKAFEPGLLAKANRGILYVDEVNLLDDHLVDVLLDSAASGWNTVERE   280
Nt_O22436.1                SGDPFNSDPDDQEVMSAEVRDKLRSGQQLPISRTKINMVDLPLGATEDRVCGTIDIEKALTEGVKAFEPGLLAKANRGILYVDEVNLLDDHLVDVLLDSAASGWNTVERE   254
Ppa_Phpat.010G011100.1     AGDPFNSSPEDPELMSEEVRKRVQANESLPVTTSRINMVDLPLGATEDRVCGTIDIEKALTEGVKAFEPGLLAKANRGILYVDEVNLLDDHLVDVLLDSAASGWNTVERE   256
Ppa_Phpat.010G011100.2     AKANRGILYVDEVNLLDDHLVDVLLDSAASGWNTVEREGISISHPARFILIGSGNPEEGELRPQLLDRFGMHAQVGTVKDAELRVKIVEERGMFDANPKSFRVNYDITQK   256
Ppa_Phpat.002G065900.1     AGDPFNSSPQDPELMSEEVRKRVQANEELPVATSRINMVDLPLGATEDRVCGTIDIEKALTEGVKAFEPGLLARANRGILYVDEVNLLDDHLVDVLLDSAASGWNTVERE   258
Cre_Cre06.g306300.t1.2     ANDPFNSDPTDPELMSEEVRNRVKAGEQLPVSSKKIPMVDLPLGATEDRVCGTIDIEKALTEGVKAFEPGLLAKANRGILYVDEVNLLDDHLVDVLLDSAASGWNTVERE   243
Vca_Vocar20008053m         ASDPFNSDPSDPELMSEEVRNRVKAGEQMSVASKKIPMVDLPLGATEDRVCGTIDIEKALTEGVKAFEPGLLAKANRGILYVDEVNLLDDHLVDVLLDSAASGWNTVERE   240
PCC6803_WP_010871795.1     ANDPFNSSPSDPEMMSEEVRIRVDSQEPLSIVKKKVTMVDLPLGATEDRVCGTIDIEKALSEGVKAFEPGLLAKANRGILYVDEVNLLDDHLVDVLLDSAAGGWNTVERE   180
Rs_AAB97156.1              EGCPVNSAR.......PEDCPEWAHVSSTTMIERPTPVVDLPLGVTEDRVVGALDIERALTRGEKAFEPGLLARANRGYLYIDEVNLLEDHIVDLLLDVAQSGENVVERE   162
Consensus                  agdpynsdp dpe mg evrerv  ge lpv  tkinmvdlplgatedrvcgtidiekaltegvkafepgllakanrgilyvdevnllddhlvdvlldsaasgwntvere
```

Continues on the next page

Figure S9. Aligned amino acid sequences of the CHLI proteins used to construct phylogenetic tree presented in Figure 1. The alignment was performed using DNAMAN program Version 6, based on sequences obtained from Phytozome v10.2 (http://phytozome.jgi.doe.gov/pz/portal.html and NCBI protein sequence database (http://www.ncbi.nlm.nih.gov/protein; protein names are indicated). The identity ≥ 50% is indicated as orange, ≥ 75% as blue, and 100% as a grey highlight. Only predicted (TargetP 1.1) chloroplast transit peptides (cTPs) are indicated by arrows, in each case the predicted cTP length is shown. Walker A and Walker B motifs are indicated by frame, the ATP binding sites are underlined, and arginine fingers are marked with ovals. The gap from position 93 to 129 in the P-loop ATPase domain in CHLI2 (transcript Cre12.g510800.t1.2) is underlined with dashed line The respective transcript names are preceded by the following coding for the species names: *Micromonas pusilla* (CCM), *Micromonas* sp. RCC299 (RCC), *Ostreococcus lucimarinus* (Olu), *Chlamydomonas reinhardtii* (Cre), *Volvox carteri* (Vca), *Coccomyxa subellipsoidea* (Csu), *Arabidopsis lyrata* (Aly), *Arabidopsis thaliana* (Ath), *Capsella grandiflora* (Cgr), *Capsella rubella* (Cru), *Boechera stricta* (Bst), *Eutrema salsugineum* (Esa), *Brassica rapa* (Bra), *Linum usitatissimum* (Lus), *Populus trichocarpa* (Ptr), *Medicago truncatula* (Mtr), *Eucalyptus grandis* (Egr), *Nicotiana tabacum* (Nt), *Physcomitrella patens* (Ppa), *Synechocystis* sp. PCC6803 (PCC6803), and *Rhodobacter sphaeroides* (Rs).

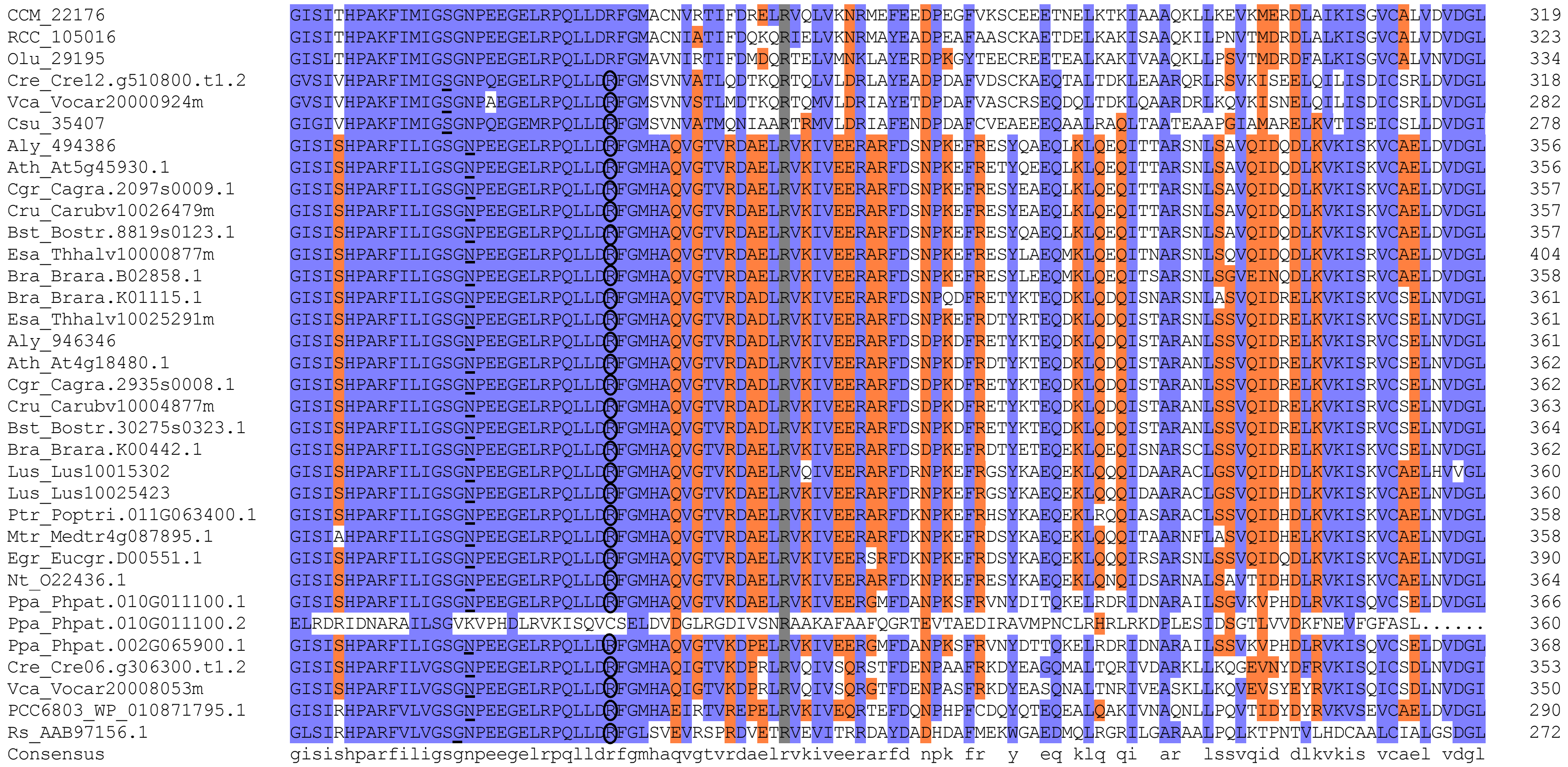

Continues on the next page

Figure S9. Aligned amino acid sequences of the CHLI proteins used to construct phylogenetic tree presented in Figure 1. The alignment was performed using DNAMAN program Version 6, based on sequences obtained from Phytozome v10.2 (http://phytozome.jgi.doe.gov/pz/portal.html and NCBI protein sequence database (http://www.ncbi.nlm.nih.gov/protein; protein names are indicated). The identity ≥ 50% is indicated as orange, ≥ 75% as blue, and 100% as a grey highlight. Only predicted (TargetP 1.1) chloroplast transit peptides (cTPs) are indicated by arrows, in each case the predicted cTP length is shown. Walker A and Walker B motifs are indicated by frame, the ATP binding sites are underlined, and arginine fingers are marked with ovals. The gap from position 93 to 129 in the P-loop ATPase domain in CHLI2 (transcript Cre12.g510800.t1.2) is underlined with dashed line The respective transcript names are preceded by the following coding for the species names: *Micromonas pusilla* (CCM), *Micromonas* sp. RCC299 (RCC), *Ostreococcus lucimarinus* (Olu), *Chlamydomonas reinhardtii* (Cre), *Volvox carteri* (Vca), *Coccomyxa subellipsoidea* (Csu), *Arabidopsis lyrata* (Aly), *Arabidopsis thaliana* (Ath), *Capsella grandiflora* (Cgr), *Capsella rubella* (Cru), *Boechera stricta* (Bst), *Eutrema salsugineum* (Esa), *Brassica rapa* (Bra), *Linum usitatissimum* (Lus), *Populus trichocarpa* (Ptr), *Medicago truncatula* (Mtr), *Eucalyptus grandis* (Egr), *Nicotiana tabacum* (Nt), *Physcomitrella patens* (Ppa), *Synechocystis* sp. PCC6803 (PCC6803), and *Rhodobacter sphaeroides* (Rs).

```
CCM_22176                   RGDIVVTRAAKALVAYERRTEVTEDDIKRVIGPCLSHRLRKDPMDTMDGSFKVMLGFNKIFKGSAMADFAGAMAEGIEDPEAKAKEEE....KAKADPAPKKAGAWG..G   423
RCC_105016                  RGDIVVTRAAKALVAYEGRDVVTEDDIKRVIGPCLSHRLRKDPLDTMDGSFKVMLGFNKVFNGSALKDFSAAMEEGVKDPEEEQRKEEEAKAKEEAAAAPKKAGAWG..G   431
Olu_29195                   RGDIVVTRAAKALVAFEGRTEVTMEDIARVIGPCLSHRLRKDVTDTMDGGFKVTLAFNKIFKGSAMLNFDETMAEGIKAPEPEKPKEA...AKPKEEPK.KKAGAWS..G   438
Cre_Cre12.g510800.t1.2      RGDIVINRAAKALVAFEGRTEVTTNDVERVISGCLNHRLRKDPLDPIDNGTKVAILFKRMTDPEIMKREEEAKKK.REEAAAKAKAEG.....KADRPTGAKAGAWA..G   420
Vca_Vocar20000924m          RGDIVINRAAKALVAFEGRAEVKLEDIERVISSCLNHRLRKDPLDPIDNGTKVKVLFKRLTDPEVQRREAEAQKA.KEEAAKKAKESGA..AAGANRPAGAKAGAWSGIG   389
Csu_35407                   RGDITTNKAARALAAFESKDTVTIDHVRRVIGLCLNHRLRKDPLETIDSGTKVALAFRRITDPQRAAKEEKAKKE.AEAAAAKAAEKA..........NKKAGAWG..G   374
Aly_494386                  RGDMVINRAARALAALQGRDQVTAEDVGIVIPNCLRHRLRKDPLESMDSGILVTEKFYKVFS................................................   418
Ath_At5g45930.1             RGDMVINRAARALAALQGRDQVTAEDVGIVIPNCLRHRLRKDPLESMDSGILVTEKFYEVFT................................................   418
Cgr_Cagra.2097s0009.1       RGDIVTNRAARALAALKGRDQVTAEDVGIVIPNCLRHRLRKDPLESMDSGILVTEKFYEVFS................................................   419
Cru_Carubv10026479m         RGDIVTNRAARALAALKGRDQVTAEDVGIVIPNCLRHRLRKDPLESMDSGILVTEKFYEVFS................................................   419
Bst_Bostr.8819s0123.1       RGDIVTNRAARALAALKGRDQVTAEDVGIVIPNCLRHRLRKDPLESMDSGILVTEKFYEVFG................................................   419
Esa_Thhalv10000877m         RGDIVTNRAARALAALKGRDQVTAEDVGIVIPNCLRHRLRKDPLESMDSGILVTEKFYEVFS................................................   466
Bra_Brara.B02858.1          RGDIVTNRAARALAALKGRDHVTAEDVGIVIPNCLRHRLRKDPLESMDSGIVVTEKFYEVFS................................................   420
Bra_Brara.K01115.1          RGDIVTNRAAKALAALKGKDRVTADDVATVIPNCLRHRLRKDPLESIDSGVLVSEKFAEVFS................................................   423
Esa_Thhalv10025291m         RGDIVTNRAAKALAALKGKDRVTADDVATVIPNCLRHRLRKDPLESIDSGVLVSEKFAEVFS................................................   423
Aly_946346                  RGDIVTNRAAKALAALKGKDRVTPDDVATVIPNCLRHRLRKDPLESIDSGVLVSEKFAEIFS................................................   423
Ath_At4g18480.1             RGDIVTNRAAKALAALKGKDRVTPDDVATVIPNCLRHRLRKDPLESIDSGVLVSEKFAEIFS................................................   424
Cgr_Cagra.2935s0008.1       RGDIVTNRAAKALAALKGKDRVTPDDVATVIPNCLRHRLRKDPLESIDSGVLVSEKFAEIFS................................................   424
Cru_Carubv10004877m         RGDIVTNRAAKALAALKGKDRVTPDDVATVIPNCLRHRLRKDPLESIDSGVLVSEKFAEIFS................................................   425
Bst_Bostr.30275s0323.1      RGDIVTNRAAKALAALKGKDRVTPDDVATVIPNCLRHRLRKDPLESIDSGVLVSEKFAEIFS................................................   426
Bra_Brara.K00442.1          RGDIVTNRAAKALAALKGKDRVTADDVATVIPNCLRHRLRKDPLESIDSGVLVSEKFAEVFS................................................   424
Lus_Lus10015302             RGDIVTNRAAKALASLKGRDKVTPEDIATVIPNCLRHRLRKDPLESIDSGLLVIEKFYEVFT................................................   422
Lus_Lus10025423             RGDIVTNRAAKALASLKGRDKVTAEDIATVIPNCLRHRLRKDPLESIDSGLLVIEKFYEVFT................................................   422
Ptr_Poptri.011G063400.1     RGDIVTNRAAKSLAALKGRDQVTAEDIATVIPNCLRHRLRKDPLESIDSGLLVIEKFYEVFS................................................   420
Mtr_Medtr4g087895.1         RGDIVTNRAAKALAGLKGINKVSAEDIATVIPNCLRHRLRKDPLESIDSGLLVIEKFYEVFT................................................   420
Egr_Eucgr.D00551.1          RGDIVSNRAAKALASLKGRDNVTLEDIATVIPNCLRHRLRKDPLESIDSGLLIIEKFYEVFT................................................   452
Nt_O22436.1                 RGDIVTNRAARALAALKGRDKVTPEDIATVIPNCLRHRLRKDPLESIDSGVLVVEKFYEVFA................................................   426
Ppa_Phpat.010G011100.1      RGDIVSNRAAKAFAAFQGRTEVTAEDIRAVMPNCLRHRLRKDPLESIDSGTLVVDKFNEVFGFAS.............................................   431
Ppa_Phpat.010G011100.2      ..............................................................................................................   360
Ppa_Phpat.002G065900.1      RGDIVSNRASKAFAAFQGRTEVTAEDIRAVMPNCLRHRLRKDPLESIDSGTLVVDKFNEVFGYST.............................................   433
Cre_Cre06.g306300.t1.2      RGDIVTNRAAKALAAFEGRTEVTPEDIYRVIPLCLRHRLRKDPLAEIDDGDRVREIFKQVFGME..............................................   417
Vca_Vocar20008053m          RGDIVTNRAAKALAAFEGRTEVTPEDIYRVIPLCLRHRLRKDPLAEIDDGDRVREVFKKVFGME..............................................   414
PCC6803_WP_010871795.1      RGDIVTNRAAKALAAFEGRTEVTVDDISRVIVLCLRHRLRKDPLESIDSGSKVEKVFKRVFGVVDEA...........................................   357
Rs_AAB97156.1               RGELTLLRAARAQAAFEGAEAVGRSHLRSVATMALSHRLRRDPLDEAGSVSRVERCVAEVLP................................................   334
Consensus                   rgdivtnraakalaalkgrd vt edv  vipnclrhrlrkdplesidsg lv ekf evf
```

Continues on the next page

Figure S9. Aligned amino acid sequences of the CHLI proteins used to construct phylogenetic tree presented in Figure 1. The alignment was performed using DNAMAN program Version 6, based on sequences obtained from Phytozome v10.2 (http://phytozome.jgi.doe.gov/pz/portal.html and NCBI protein sequence database (http://www.ncbi.nlm.nih.gov/protein; protein names are indicated). The identity ≥ 50% is indicated as orange, ≥ 75% as blue, and 100% as a grey highlight. Only predicted (TargetP 1.1) chloroplast transit peptides (cTPs) are indicated by arrows, in each case the predicted cTP length is shown. Walker A and Walker B motifs are indicated by frame, the ATP binding sites are underlined, and arginine fingers are marked with ovals. The gap from position 93 to 129 in the P-loop ATPase domain in CHLI2 (transcript Cre12.g510800.t1.2) is underlined with dashed line The respective transcript names are preceded by the following coding for the species names: *Micromonas pusilla* (CCM), *Micromonas* sp. RCC299 (RCC), *Ostreococcus lucimarinus* (Olu), *Chlamydomonas reinhardtii* (Cre), *Volvox carteri* (Vca), *Coccomyxa subellipsoidea* (Csu), *Arabidopsis lyrata* (Aly), *Arabidopsis thaliana* (Ath), *Capsella grandiflora* (Cgr), *Capsella rubella* (Cru), *Boechera stricta* (Bst), *Eutrema salsugineum* (Esa), *Brassica rapa* (Bra), *Linum usitatissimum* (Lus), *Populus trichocarpa* (Ptr), *Medicago truncatula* (Mtr), *Eucalyptus grandis* (Egr), *Nicotiana tabacum* (Nt), *Physcomitrella patens* (Ppa), *Synechocystis* sp. PCC6803 (PCC6803), and *Rhodobacter sphaeroides* (Rs).

CCM_22176	LPG.G	427
RCC_105016	LPGFG	436
Olu_29195	LPG.G	442
Cre_Cre12.g510800.t1.2	LPPRR	425
Vca_Vocar20000924m	LPSRR	394
Csu_35407	LPGPK	379
Aly_494386	.....	418
Ath_At5g45930.1	.....	418
Cgr_Cagra.2097s0009.1	.....	419
Cru_Carubv10026479m	.....	419
Bst_Bostr.8819s0123.1	.....	419
Esa_Thhalv10000877m	.....	466
Bra_Brara.B02858.1	.....	420
Bra_Brara.K01115.1	.....	423
Esa_Thhalv10025291m	.....	423
Aly_946346	.....	423
Ath_At4g18480.1	.....	424
Cgr_Cagra.2935s0008.1	.....	424
Cru_Carubv10004877m	.....	425
Bst_Bostr.30275s0323.1	.....	426
Bra_Brara.K00442.1	.....	424
Lus_Lus10015302	.....	422
Lus_Lus10025423	.....	422
Ptr_Poptri.011G063400.1	.....	420
Mtr_Medtr4g087895.1	.....	420
Egr_Eucgr.D00551.1	.....	452
Nt_O22436.1	.....	426
Ppa_Phpat.010G011100.1	.....	431
Ppa_Phpat.010G011100.2	.....	360
Ppa_Phpat.002G065900.1	.....	433
Cre_Cre06.g306300.t1.2	.....	417
Vca_Vocar20008053m	.....	414
PCC6803_WP_010871795.1	.....	357
Rs_AAB97156.1	.....	334
Consensus		

Identity = 83.02% **= 100%** **≥ 75%** **≥ 50%**

Figure S9. Aligned amino acid sequences of the CHLI proteins used to construct phylogenetic tree presented in Figure 1. The alignment was performed using DNAMAN program Version 6, based on sequences obtained from Phytozome v10.2 (http://phytozome.jgi.doe.gov/pz/portal.html and NCBI protein sequence database (http://www.ncbi.nlm.nih.gov/protein; protein names are indicated). The identity ≥ 50% is indicated as orange, ≥ 75% as blue, and 100% as a grey highlight. Only predicted (TargetP 1.1) chloroplast transit peptides (cTPs) are indicated by arrows, in each case the predicted cTP length is shown. Walker A and Walker B motifs are indicated by frame, the ATP binding sites are underlined, and arginine fingers are marked with ovals. The gap from position 93 to 129 in the P-loop ATPase domain in CHLI2 (transcript Cre12.g510800.t1.2) is underlined with dashed line The respective transcript names are preceded by the following coding for the species names: *Micromonas pusilla* (CCM), *Micromonas* sp. RCC299 (RCC), *Ostreococcus lucimarinus* (Olu), *Chlamydomonas reinhardtii* (Cre), *Volvox carteri* (Vca), *Coccomyxa subellipsoidea* (Csu), *Arabidopsis lyrata* (Aly), *Arabidopsis thaliana* (Ath), *Capsella grandiflora* (Cgr), *Capsella rubella* (Cru), *Boechera stricta* (Bst), *Eutrema salsugineum* (Esa), *Brassica rapa* (Bra), *Linum usitatissimum* (Lus), *Populus trichocarpa* (Ptr), *Medicago truncatula* (Mtr), *Eucalyptus grandis* (Egr), *Nicotiana tabacum* (Nt), *Physcomitrella patens* (Ppa), *Synechocystis* sp. PCC6803 (PCC6803), and *Rhodobacter sphaeroides* (Rs).
